# Structural and evolutionary bioinformatics of the SPOUT superfamily of methyltransferases

**DOI:** 10.1186/1471-2105-8-73

**Published:** 2007-03-05

**Authors:** Karolina L Tkaczuk, Stanislaw Dunin-Horkawicz, Elzbieta Purta, Janusz M Bujnicki

**Affiliations:** 1 Laboratory of Bioinformatics and Protein Engineering, International Institute of Molecular and Cell Biology in Warsaw, Trojdena 4 St., PL-02-109 Warsaw, Poland; 2 Institute of Technical Biochemistry, Faculty of Biotechnology and Food Sciences, Technical University of Lodz, Stefanowskiego 4/10 St., PL- 90-924 Lodz, Poland; 3 Institute of Biochemistry and Biophysics, Polish Academy of Sciences, Pawinskiego 5a St., PL-02-106 Warsaw, Poland; 4 Institute of Molecular Biology and Biotechnology, Adam Mickiewicz University, Umultowska 89, PL-61-614 Poznan, Poland

## Abstract

**Background:**

SPOUT methyltransferases (MTases) are a large class of S-adenosyl-L-methionine-dependent enzymes that exhibit an unusual alpha/beta fold with a very deep topological knot. In 2001, when no crystal structures were available for any of these proteins, Anantharaman, Koonin, and Aravind identified homology between SpoU and TrmD MTases and defined the SPOUT superfamily. Since then, multiple crystal structures of knotted MTases have been solved and numerous new homologous sequences appeared in the databases. However, no comprehensive comparative analysis of these proteins has been carried out to classify them based on structural and evolutionary criteria and to guide functional predictions.

**Results:**

We carried out extensive searches of databases of protein structures and sequences to collect all members of previously identified SPOUT MTases, and to identify previously unknown homologs. Based on sequence clustering, characterization of domain architecture, structure predictions and sequence/structure comparisons, we re-defined families within the SPOUT superfamily and predicted putative active sites and biochemical functions for the so far uncharacterized members. We have also delineated the common core of SPOUT MTases and inferred a multiple sequence alignment for the conserved knot region, from which we calculated the phylogenetic tree of the superfamily. We have also studied phylogenetic distribution of different families, and used this information to infer the evolutionary history of the SPOUT superfamily.

**Conclusion:**

We present the first phylogenetic tree of the SPOUT superfamily since it was defined, together with a new scheme for its classification, and discussion about conservation of sequence and structure in different families, and their functional implications. We identified four protein families as new members of the SPOUT superfamily. Three of these families are functionally uncharacterized (COG1772, COG1901, and COG4080), and one (COG1756 represented by Nep1p) has been already implicated in RNA metabolism, but its biochemical function has been unknown. Based on the inference of orthologous and paralogous relationships between all SPOUT families we propose that the Last Universal Common Ancestor (LUCA) of all extant organisms contained at least three SPOUT members, ancestors of contemporary RNA MTases that carry out m^1^G, m3U, and 2'O-ribose methylation, respectively. In this work we also speculate on the origin of the knot and propose possible 'unknotted' ancestors. The results of our analysis provide a comprehensive 'roadmap' for experimental characterization of SPOUT MTases and interpretation of functional studies in the light of sequence-structure relationships.

## Background

SPOUT methyltransferases (MTases) [1] are a large class of S-adenosyl-L-methionine (AdoMet)-dependent enzymes that exhibit an unusual fold with a very deep topological knot [2,3]. Historically, it has been the 4^th ^unrelated type of AdoMet-dependent MTase structure determined by X-ray crystallography, hence named 'class IV' (review: [4]). SPOUT homologs are present in multiple copies in all proteomes [1,5] and among AdoMet-dependent MTases are outnumbered only by the class I, Rossmann-fold MTases (RFM) [6,7]. Nonetheless, only a few SPOUT members have been characterized functionally. Thus far, all of them have been found to be involved in posttranscriptional RNA modification and introduce methylation of 2'-OH groups of ribose (in tRNA – Trm3 [8], TrmH [9], or rRNA – RlmB [3]), or the N-1 atom of guanosine 37 in tRNA (TrmD, [10,11]), or the N-3 atom of uridine 1498 in 16S rRNA (RsmE; [12]). During the last few years, a number of crystal structures solved by the structural genomics initiative revealed a common fold of SPOUT MTases, the so-called 'α/β knot fold', also in members of uncharacterized protein families. Currently (January 2007), the majority of members of this fold listed in the SCOP database [13] are proteins with uncharacterized function, and it is likely that this fraction will continue to grow with the progress of structural genomics. For simplicity, in this work each domain with the 'α/β knot' fold, determined experimentally or predicted computationally, will be referred to as the SPOUT domain. All evidence suggests that *bona fide *SPOUT MTases and structurally similar but functionally uncharacterized proteins are homologous and should be classified within the same superfamily. Consequently, the whole group of proteins with the SPOUT domain will be referred to as the 'SPOUT superfamily', regardless of the level of their functional characterization and degree of sequence similarity to previously identified SPOUT MTases.

Among the few experimental studies on SPOUT enzymes, two focused on characterization of potential active sites identified in the structures of TrmH [14,15] and TrmD [11], providing insight into the role of amino acids conserved within these two families of SPOUT MTases. However, since the pioneering sequence analysis of Anantharaman et al., which was done before any structures of SPOUT MTases were available [1], no comprehensive comparative analysis of sequence conservation between these proteins and other (uncharacterized) members was carried out. Thus, molecular determinants of different catalytic mechanisms in other SPOUT MTases remain completely unknown. Without this knowledge, it is very difficult to make predictions about the molecular function of experimentally uncharacterized members of the SPOUT superfamily, including those with structures solved by structural genomics. It is also tempting to speculate that other protein families without available structures and with no obvious sequence similarity to known members may belong to the SPOUT superfamily and still await discovery. Finally, the evolutionary origin of this strongly conserved fold, and especially its deep topological knot, is unknown.

Motivated by the scale of what is unknown about the SPOUT superfamily, we carried out a search for all its members, followed by comprehensive sequence and structure analyses. The aim of this study was to provide a structural and evolutionary classification of SPOUT proteins that will serve as a guide for analyses of sequence-structure-function relationships, and to make structural and functional predictions for individual proteins that will stimulate their experimental characterization.

## Results

### Structural features of known members of the SPOUT superfamily

We collected all crystal structures of known members of the SPOUT superfamily annotated in the SCOP database [13] as the 'α/β knot superfamily'. These structures were used to search the Protein Data Bank (PDB) with DALI [16] and SSM [17] to identify additional proteins with similar topology. The resulting dataset was purged to retain only one structure per protein (preference was given to native proteins over ones with selenomethionine, and to those with the highest resolution) yielding 19 non-redundant protein structures, including 2ha8, 1zjr and 1x7o that were not present in SCOP. Table 1 shows DALI scores describing their mutual similarities.

**Table 1:** Similarities Between all SPOUT Structures Detectable With DALI

PDB id	name	organism	specificity	family	1v2x	1zjr	2ha8	1gz0	1ipa	1x7o	1j85	1nxz	1vhk	1v6z	1z85	1k3r	1ns5	1to0	1vh0	1o6d	1oy5	1p9p	1uaj
1v2x	TrmH	*T. thermophilus*	tRNA:Gm18	COG0566A	X	**21.4**	**21**	**22.5**	**21.7**	**20.8**	**16.2**	**11.8**	**12.9**	**17.7**	10.3	**13.2**	9.1	8.8	8.7	8.2	7.2	8.3	7.4
1zjr	TrmH	*A. aeolicus*	tRNA:Gm18	COG0566A	**21.4**	X	**19.3**	**22.3**	**17.7**	**17.6**	**14.5**	10.7	**11**	**12.2**	9.1	**11.1**	7.8	7.9	7.4	7.4	5.5	6.4	6.3
2ha8	HTRBP1	*H. sapiens*	tRNA:Gm18?	COG0566A	**21**	**19.3**	X	**20.3**	**19.2**	**20.5**	**17.7**	**12.4**	**12.9**	**13**	10.2	**10.9**	8.6	9	8.5	9.1	7.8	8.9	8.9
1gz0	RlmB	*E. coli*	23S rRNA:Gm2251	COG0566B	**22.5**	**22.3**	**20.3**	X	**23.6**	**22.3**	**17.3**	**12.3**	**11.5**	**13.8**	**11.2**	**13.3**	8.3	8.2	7.9	7.9	6.6	7.2	6.7
1ipa	RrmA	*T. thermophilus*	unknown	COG0566C	**21.7**	**17.7**	**19.2**	**23.6**	X	**20.5**	**17.7**	**12.7**	**12.1**	**13.1**	**11.1**	**13.4**	9.5	9.2	9	8	6.4	7.3	5.9
1x7o	AviRb	*S. viridochromogenes*	23S rRNA:Um2479	COG0566C	**20.8**	**17.6**	**20.5**	**22.3**	**20.5**	X	**17.9**	**11.7**	**12.4**	**13.4**	10.2	**12.1**	8.2	8.4	7.8	7.8	6.5	7.9	7.8
1j85	YibK	*H. influenzae*	unknown	COG0219	**16.2**	**14.5**	**17.7**	**17.3**	**17.7**	**17.9**	X	10.8	**12.3**	**13**	10.3	**11.7**	7.1	7.9	7.1	8.5	5.9	6.8	6.9
1nxz	RsmE	*H. influenzae*	16S rRNA:m3U1498?	COG1385	**11.8**	10.7	**12.4**	**12.3**	**12.7**	**11.7**	10.8	X	**22.8**	**21**	**19.4**	**13.9**	9.3	8.3	8.5	9.7	6.2	7.3	7.1
1vhk	YqeU	*B. subtilis*	16S rRNA:m3U1498?	COG1385	**12.9**	**11**	**12.9**	**11.5**	**12.1**	**12.4**	**12.3**	**22.8**	X	**23**	**17.5**	**12.8**	9	8.9	8.1	8.7	6.4	7.7	7.4
1v6z	TT1573	*T. thermophilus*	16S rRNA:m3U1498?	COG1385	**13.8**	**12.2**	**13**	**13.8**	**13.1**	**13.4**	**13**	**21**	**23**	X	**14.3**	**13.8**	8.3	8.4	7.3	7.7	5.8	7	6.8
1z85	TM1380	*T. maritima*	16S rRNA:m3U1498?	COG1385	10.3	9.1	10.2	**11.2**	**11.1**	10.2	10.3	**19.4**	**17.5**	**14.3**	X	**11.2**	5.2	5.9	5	5.9	5.9	5.9	4.9
1k3r	MT1	*M. thermoautotrophicum*	unknown	COG2106	**13.2**	**11.1**	**10.9**	**13.3**	**13.4**	**12.1**	**11.5**	**13.9**	**12.8**	**13.8**	**11.2**	X	10.6	10.2	8.5	9.7	7.4	7.9	7.7
1ns5	YbeA	*E. coli*	unknown	COG1576	9.1	7.8	8.6	8.3	9.5	8.2	8.3	9.3	9	8.3	5.2	10.6	X	**23.8**	**21.9**	**22.7**	9.6	**11.1**	10.1
1to0	sr145	*B. subtilis*	unknown	COG1576	8.8	7.9	9	8.2	9.2	8.4	7.9	8.3	8.9	8.4	5.9	10.2	**23.8**	X	**26.4**	**22.4**	10.2	**11**	10.8
1vh0	SAV0024	*S. aureus*	unknown	COG1576	8.7	7.4	8.5	7.9	9	7.8	7.1	8.5	8.1	7.3	5	8.5	**21.9**	**26.4**	X	**22.8**	10.5	**11.2**	9.6
1o6d	TM0844	*T. maritima*	unknown	COG1576	8.2	7.4	9.1	7.9	8	7.8	8.5	9.7	8.7	7.7	5.9	9.7	**22.7**	**22.4**	**22.8**	X	10.3	**11.6**	**11.9**
1oy5	TrmD	*A. aeolicus*	tRNA:m1G37	COG0336	7.2	5.5	7.8	6.6	6.4	6.5	5.9	6.2	6.4	5.8	5.9	7.4	9.6	10.2	10.5	10.3	X	**21.1**	**20.6**
1p9p	TrmD	*E. coli*	tRNA:m1G37	COG0336	8.3	6.4	8.9	7.2	7.3	7.9	6.8	7.3	7.7	7	5.9	7.9	**11.1**	**11**	**11.2**	**11.6**	**21.1**	X	**29.1**
1uaj	TrmD	*H. influenzae*	tRNA:m1G37	COG0336	7.4	6.3	8.9	6.7	5.9	7.8	6.9	7.1	7.4	6.8	4.9	7.7	10.1	10.8	9.6	**11.9**	**20.6**	**29.1**	X

Structures are described by their PDB IDs. Pairwise structural similarities according to the DALI Z-score (number of standard deviations above the expectation for unrelated structures) are listed, highest values are indicated by black (Z ≥ 15) or grey background (15 > Z ≥ 11). The highest similarity (black shading) correlates very well with membership in different SCOP families within the "α/β knot superfamily".

Subsequently, we assigned SPOUT structures to sequence alignments of previously identified SPOUT-related families: SpoU, Aq_054, AF2226, TrmD, YbeA [1], and DUF431 [18]. Structures with no matches to these 'old' SPOUT families were regarded as founding members of new families and used to identify the respective alignments in the COG (Clusters of Orthologous Groups) database using PSI-BLAST. For families with structurally characterized representatives, secondary structure was assigned to the alignment according to DSSP. For the remaining previously known SPOUT families (COG0336, COG0565, COG1303, COG1385, COG1818, COG2419, COG2428, and COG4752) we carried out secondary and tertiary structure prediction and built comparative models (see Methods) and assigned the secondary structure to the alignments based on the predictions.

**Figure 1:**
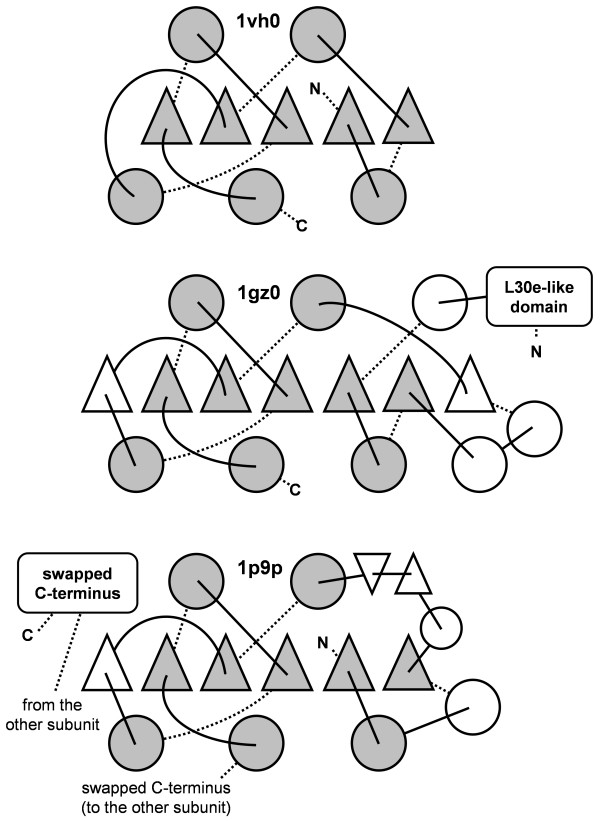
**Conserved topology of the common core and the most typical architecture of the active site in SPOUT proteins, exemplified by a 'minimal' putative MTase SAV0024 (1vh0), RlmB (1gz0), and TrmD (1p9p)**. Helices are shown as circles, strands are shown as triangles. Universally conserved elements are shown in grey, variable elements are in white.

Comparison of SPOUT domains reveals the presence of a common core, comprising a parallel β-sheet of 5 strands, sandwiched between two layers of helices (Figure 1). The SPOUT domain can be subdivided into the variable N-terminal subdomain with a Rossmanoidal α/β fold, and the conserved C-terminal trefoil knot (also exhibiting the α/β structure) that binds AdoMet. The structure of a putative MTase SAV0024/SA0023 from *Staphylococcus aureus *(1vh0 in the Protein Data Bank, PDB) is an example of a SPOUT member that contains only the core elements. Even in the universally conserved core the helices vary greatly in size and angle with respect to each other and to the β-sheet and often cannot be simultaneously superimposed if strongly diverged members are compared (data not shown). In many members (e.g. TrmH, RlmB, RsmE) the core is extended by addition of one α/β module to the N-terminal subdomain (right edge of the β-sheet in Figure 1). On the other hand, the α/β module on the other side of the β-sheet shows significant variability in the content of regular secondary structure, despite topological conservation, resulting in apparent 'melting' of a β-strand into a loop or shrinking of the α-helix (Figure 1).

**Figure 2:**
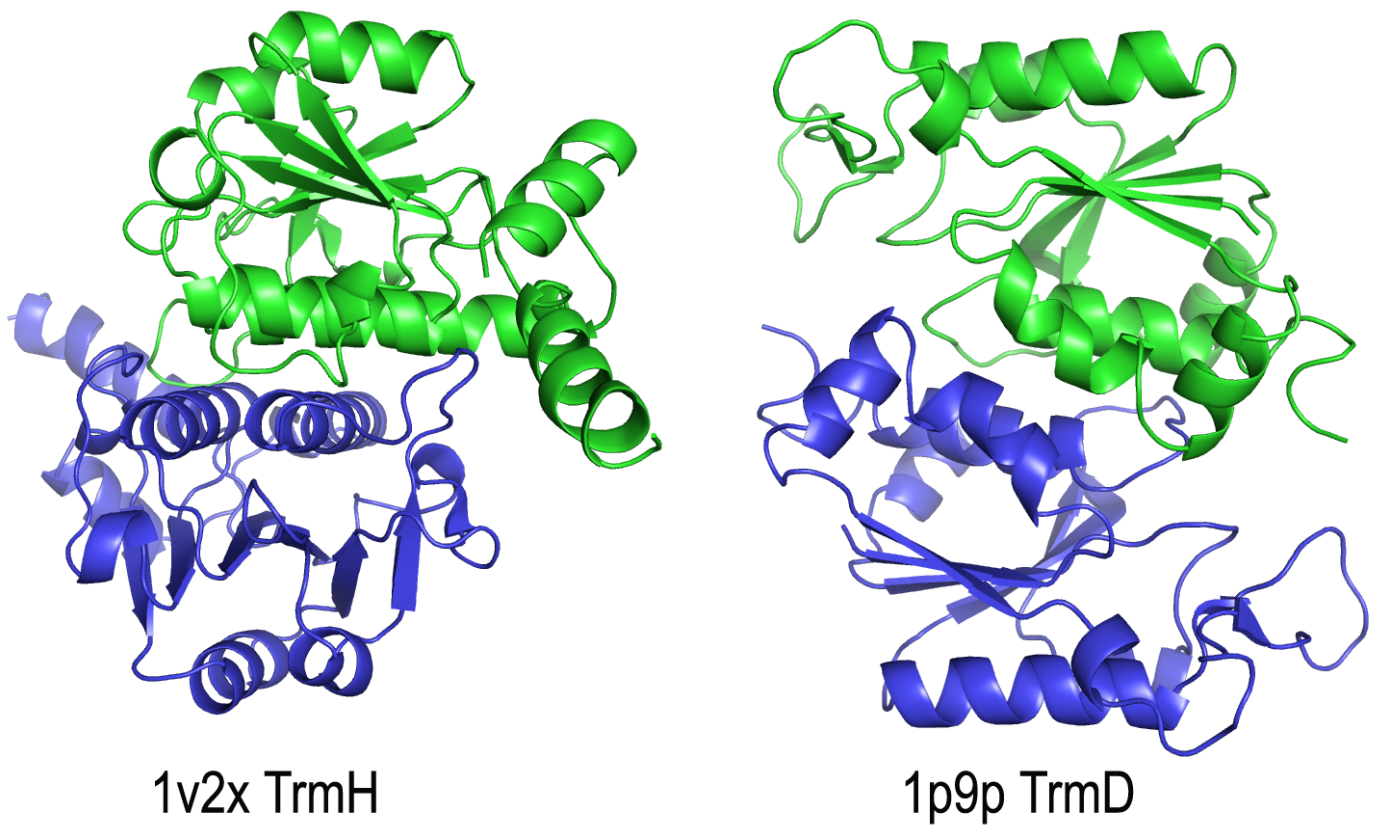
**'Perpendicular' and 'antiparallel' modes of dimerization observed among SPOUT enzymes, exemplified by TrmH (1v2x, left) and TrmD (1p9p, right)**. One monomer (indicated in green, shown on the top) is shown in the same orientation in both proteins, while the other one (indicated in blue, shown on the bottom) is rotated by about 90 degrees (TrmH) or 180 degrees (TrmD) with respect to the first one.

All SPOUT members studied to date were found to be dimers, with a large surface area buried upon dimerization. In all structures, the cofactor-binding loop in the knot of one monomer is stabilized by interactions with the other monomer. Moreover, the active sites are formed by residues from both monomers, suggesting that dimerization is essential for the MTase activity. Interestingly, different SPOUT structures exhibit different modes of dimerization. For instance, in the dimers formed by RlmB, TrmH, RrmA, YibK, RsmE, and MT1, the two sheets are oriented in a nearly perpendicular way, while in TrmD and YbeA they are antiparallel (Figure 2.)

**Figure 3:**
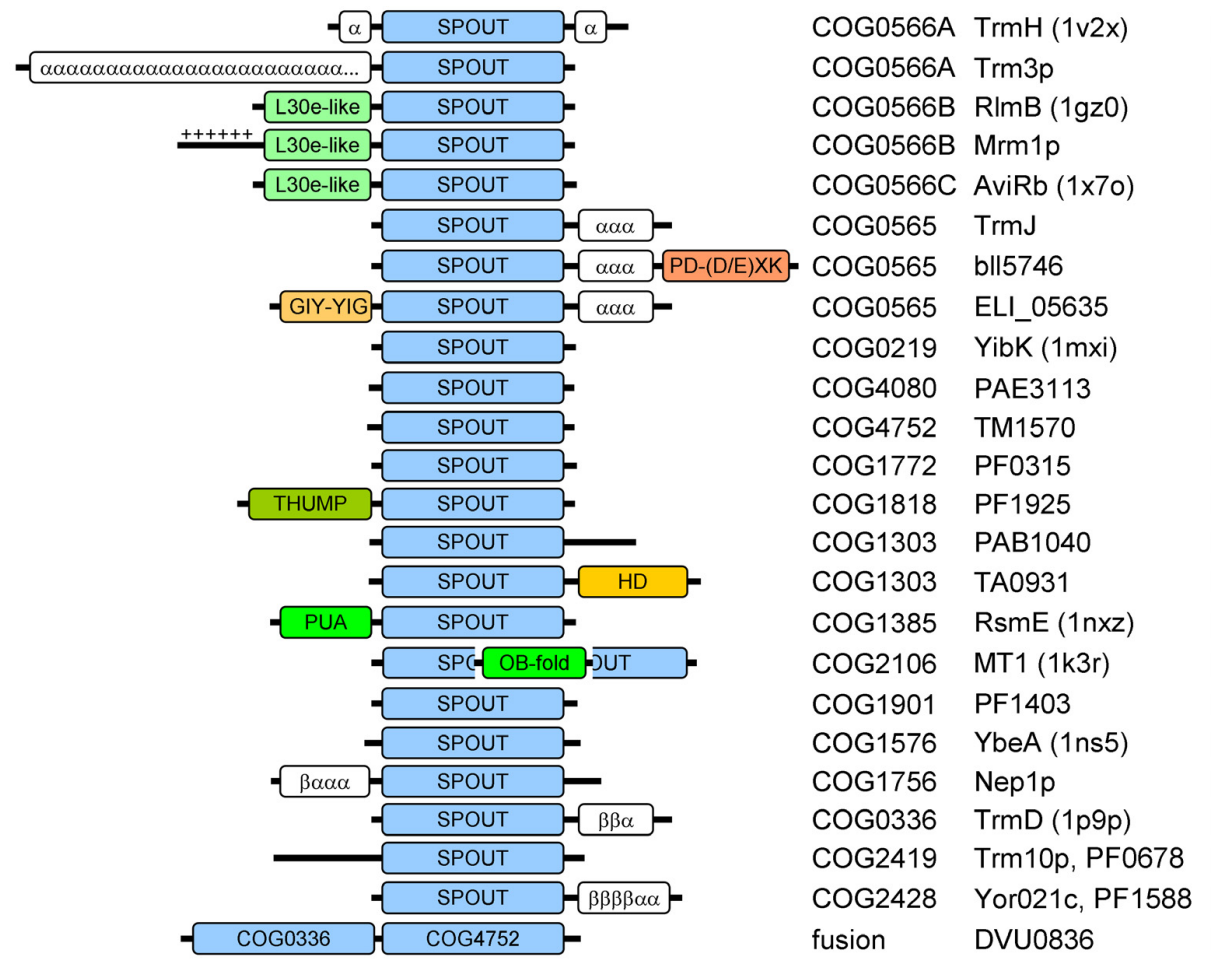
**Domain architectures observed in the SPOUT superfamily**. Light blue blocks indicate the common catalytic domain. Other known domains are shown in different colors. Uncharacterized extensions, which may or may not form independent domains, are indicated as white boxes with the corresponding patterns of predicted secondary structure (α helices and β strands). In the example shown at the bottom, a protein comprising two SPOUT domains from different families is shown.

Many SPOUT members exhibit additional domains fused to the N- or C-termini or inserted into a linker between the two subdomains (Figure 3). Interestingly, most of these domains belong to various superfamilies of common nucleic acid binding domains, such as PUA, THUMP, OB-fold, or L30e [19-21], or hydrolases implicated in metabolism of nucleic acids, such as the PD-(D/E)XK nuclease superfamily [22], the GIY-YIG nuclease superfamily [23], or the HD metal-dependent phosphohydrolase superfamily [24]. These domain fusions strongly suggest that the SPOUT superfamily is involved primarily in binding and enzymatic modification of nucleic acids, unlike other MTase superfamilies that are either very promiscuous or specialize in methylation of proteins or small molecules [4,5].

### Identification of new members of the SPOUT superfamily

We used HHsearch [25] to convert alignments of the known SPOUT families into profile-Hidden Markov Models (HMMs) that include both the sequences and secondary structure, and we used them to identify other potentially homologous families. The HHpred method for HMM-HMM comparisons was used to search a database of profile-HMMs corresponding to multiple sequence alignments obtained from the COG, KOG, and PFAM databases, also with predicted secondary structures. Based on the results of the HMM-HMM comparison, and in particular the list of 'hits' with significant P-value (< 0.0001) and scores (>15.0), we generated a preliminary list of new candidate SPOUT families. These parameters were chosen to detect as many protein families with remote similarity to SPOUT as possible, including possibly all true homologs that had to be confirmed by more refined analyses, as well as unavoidable false positives that had to be removed in subsequent steps of the analysis.

The preliminary candidates were initially validated by reciprocal HHsearches against the database comprising both the initial query profile-HMMs as well as all the other COG, KOG, and PFAM profile HMMs. If a region of sequence that initially seemed to be similar to known SPOUT members displayed evident strong similarity to some other family, known to be unrelated to SPOUT, then a given family was regarded as a false positive and removed from the list of SPOUT candidates. Each candidate family, for which the relationship to known SPOUTs was confirmed by reciprocal searches, was also analyzed by fold-recognition (FR) methods via the GeneSilico metaserver [26]. This step served to test whether the sequence of the new candidate is compatible with the 'α/β knot' fold or with some other unrelated structure. Finally, comparative models were built for the parts of the sequence aligned by the FR methods to the template structures and the sequence conservation was analyzed in the structural context to detect potential active site residues.

Ultimately, our analysis revealed that so far uncharacterized (or poorly characterized) and structurally unclassified families COG1756 (archaeal and eukaryotic proteins represented by Nep1p), COG1772 (archaeal proteins represented by PF0315), COG1901 (archaeal proteins represented by PF1403), and COG4080 (archaeal protein represented by MJ1199), are related to SPOUT MTases and share the characteristic "α/β knot" structure. Below we report the supporting evidence for the SPOUT fold prediction obtained from the FR analysis. Detailed structural predictions and the resulting functional implications for these new members of the SPOUT superfamily will be described in the following sections devoted to evolutionary analyses.

#### COG1756 (representative: Nep1p)

The profile-HMM analysis confidently identified a relationship (P-value 1.4*10^-4^) between the C-terminal domain of COG1756 (aa 150–298) and the TrmD family (COG0366), as well as other SPOUT families, albeit with lower scores. The FR analysis identified SPOUT structures, mainly 1o6d and 1ns5 from the YbeA family (COG1576), as the best templates for modeling of COG1756 sequences, with good agreement of alignments reported by different servers despite moderate scores (mGenTHREADER: score 0.436, SAM-T02: score 0.56, 3DPSSM: score: 0.7). The consensus predictor PCONS selected 1o6d as a preferred template (score 1.427).

#### COG1772 (PF04407, DUF531; representative: PF0315)

The HHsearch analysis carried out for COG1772 identified its relationship to COG4752 (TM1570) and COG0565 (TrmJ family) with P-values: 1.3*10^-4 ^and 4.5*10^-4 ^respectively, and a more remote similarity to COG0566 (P-value 1.3*10^-3^). The FR analysis identified the SPOUT domain of COG0566 members: RlmB (1gz0), RrmA (1ipa), and AviRb (1x7o) as the potentially best modeling templates for COG1772 sequences, although with relatively low scores (FFAS: -7.79, mGenTHREADER: 0.588, SPARKS: -3.14, consensus server PCONS – top 4 positions, score 1.045–1.4837). Importantly, COG1772 proteins lack the L30e-like N-terminal domain common to these preferred templates and therefore comprises only the SPOUT domain.

#### COG4080 (representatives: MJ1199, APE2001)

The profile-HMM comparison revealed a close relationship of COG4080 to COG0565 (probability: 93.2%, P-value: 3.3*10^-7^) and slightly more remote connection to COG0566, COG0219, and COG1303 (probability > 82% and P-values ~ 10^-5^). The FR analysis carried out for COG4080 identified structures of COG0566 proteins as the best templates, even though COG4080 members lack the L30e-like domain. The best-scoring templates were: 1gz0 (INBGU: 139.24, mGenTHREADER: 0.687, FFAS: -15.6). 1v2x (INBGU: 139.69, 3DPSSM: 0.058, FFAS: -13.4), and 1j85 (INBGU: 127.32, mGenTHREADER: 0.675, FFAS: -17.9. These results suggest that the previous annotation of COG4080 "RecB-family nuclease [DNA replication, recombination, and repair]" is probably spurious, as these proteins comprise exclusively the SPOUT domain and are definitely unrelated to the RecB family.

#### COG1901 (PF04013; DUF358), representative: PF1403)

The HHsearch analysis revealed that COG1901 displays close relationships to COG1756 (probability: 88.8% and P-value:5.3*10^-6^) and COG1385 (probability: 80.6% and P-value:1.1*10^-5^). Most FR servers identified 1j85 (COG0219) as the best template with high scores (SPARKS: -2.53, mGenTHREADER: 0.476, consensus servers Pcons2: 0.719 and Pcons5: 0,829). Other highly scored templates belonged to COG1385 (1vhk/1vxz/1vhy) INBGU: 36.02 SAM-T02: 0.015, SPARKS: -2.52, FUGUE: 3.92.

We used the newly identified SPOUT families as queries to carry out additional profile-HMM searches, but no new families could be identified to show similarity to the expanded SPOUT dataset. We observed no new relationships even when all (old and new) profiles were enriched with additional sequences identified in PSI-BLAST searches (see below). This suggests that we obtained a comprehensive representation of the SPOUT superfamily and if any homologous families remain undetected, they must exhibit extreme divergence from sequences analyzed in this work.

### Clustering analysis of the SPOUT superfamily

To identify a complete set of SPOUT sequences (beyond those included in the COG or PFAM alignments), we used representatives of all families to carry out exhaustive PSI-BLAST searches of the nr database (until convergence) and retrieved all sequences reported with e-value < 10^-3^. After removing obvious duplicates (identical proteins retrieved in different searches), we obtained a set of over 1900 sequences. Because such a large number of sequences are very difficult to handle, we decided to generate a preliminary classification and refine the alignments independently for each family, and only then to carry out the evolutionary analysis. Clustering of all full-length SPOUT sequences was performed based on their pair-wise BLAST similarity scores, using CLANS [27]. We have experimentally found that for this particular dataset the P-value threshold of 10^-3 ^produced qualitatively best results (more stringent values caused disconnection of the most diverged families, while more permissive values caused over-compaction of the whole dataset into a single cluster with only a few outliers). This simple clustering was remarkably effective, as it produced a clear-cut separation of most original COGs and their close homologs found by PSI-BLAST into 18 distinct clusters. Based on the results of preliminary clustering, we extracted members of individual families and calculated multiple sequence alignments using MUSCLE [28] and refined them manually (as described in Methods) to remove truncated sequences and redundant, nearly identical versions of the same protein, and to improve the placement of insertions and deletions. We used these refined alignments to re-scan the database of profile-HMMs using HHSearch, but the results were qualitatively similar to those obtained with the original alignments obtained from the COG and PFAM database (e.g. the same set of SPOUT families were identified, with similar scores).

**Figure 4:**
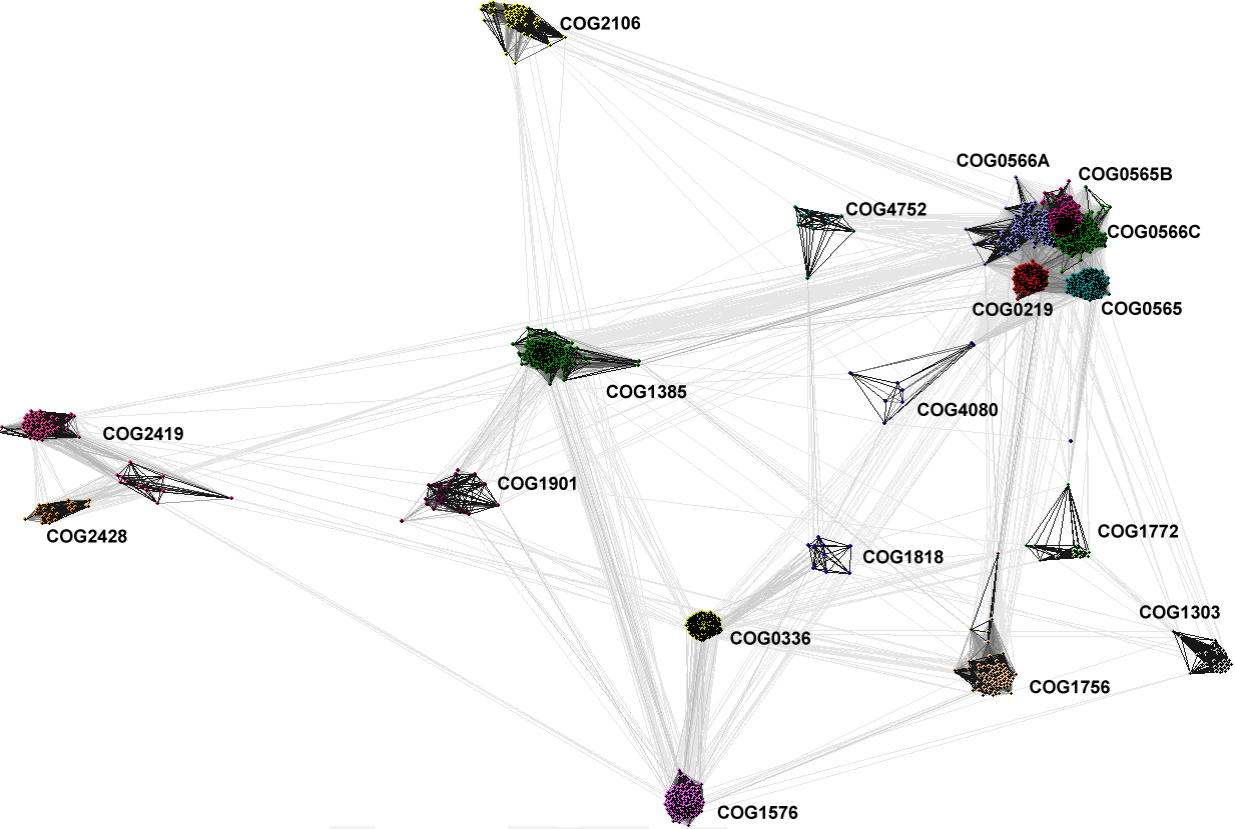
**Two-dimensional projection of the CLANS clustering results obtained for the full-length SPOUT sequences**. Proteins are indicated by dots, colored according to the membership in different families (COGs). Lines indicate sequence similarity detectable with BLAST and are colored by a spectrum of shades of grey according to the BLAST P-value (black: P-value < 10^-200^, light grey: P-value < 10^-5^). Significant differences in the size of families could distort the results of clustering. Thus, for all families of size >50 we used the PURGE option of the Gibbs motif sampler [84] to identify 50 representative sequences with the maximal sequence divergence. However, the results of CLANS analyses for this 'representative' set of SPOUT members were very similar to those obtained for the full dataset (data not shown).

**Figure 5:**
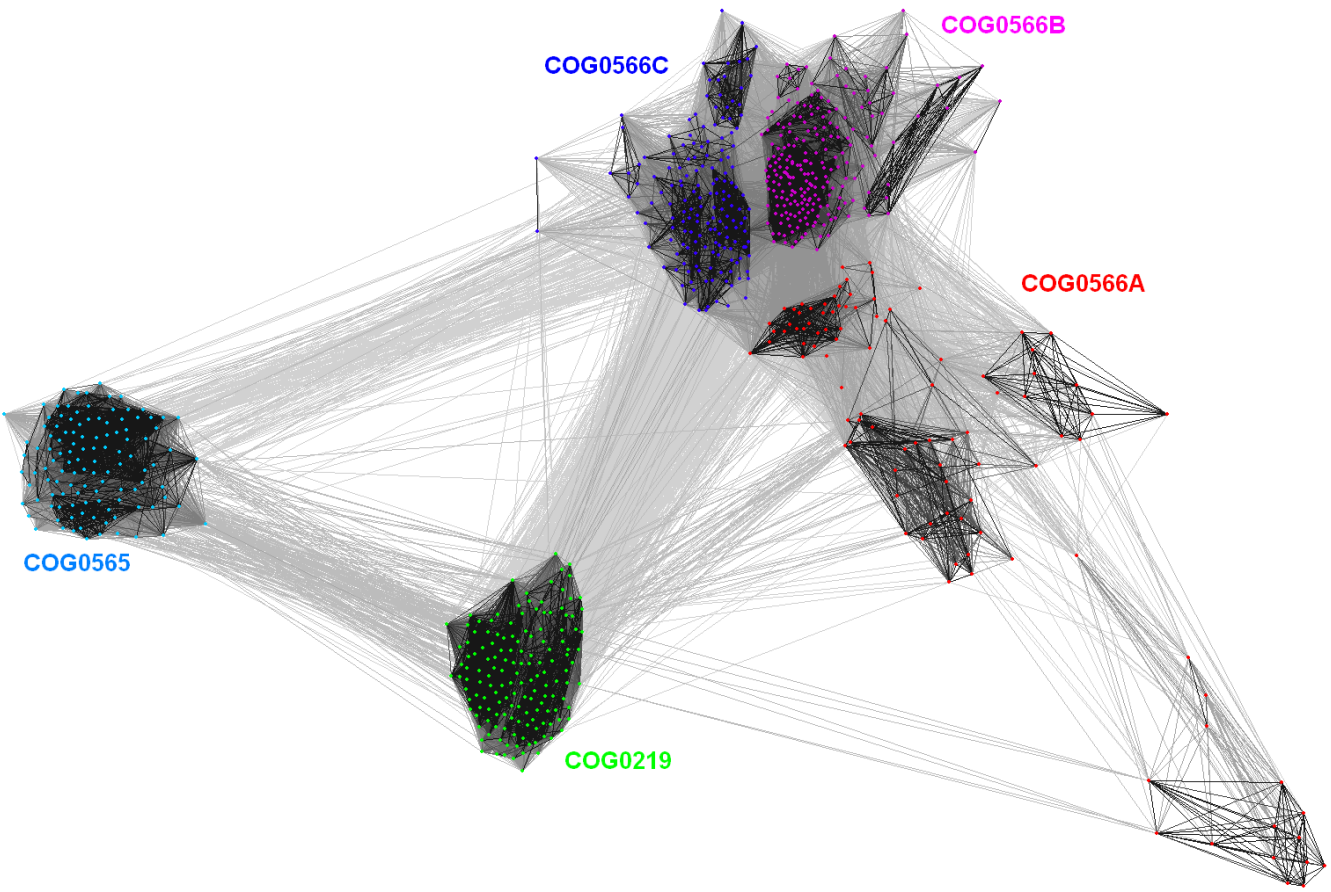
**Two-dimensional projection of the CLANS clustering results obtained for the full-length sequences of the 'supercluster'**. Proteins are indicated by colors according to their membership in families and subfamilies. Lines indicate sequence similarity detectable with BLAST and are colored by a spectrum of shades of grey according to the BLAST P-value (black: P-value < 10^-45^, light grey: P-value < 10^-5^).

While refining the alignment of TrmD homologs (COG0336), we discovered that three sequences (DVU0836 from *Desulfovibrio vulgaris*, LI0220 from *Lawsonia intracellularis*, and MldDRAFT_4740 from Deltaproteobacterium MLMS-1) comprise a fusion of the TrmD-like domain with a second SPOUT domain that belongs to COG4752 (Figure 3). In order to avoid artificial bridging of COG0336 and COG4752 by these fusion proteins, their sequences were split into two separate parts and the clustering of the SPOUT superfamily was repeated (Figure 4). Subsequently, we carried out a separate analysis for the largest central supercluster, which groups together strongly interconnected members of COG565, COG0566, and COG0219. At the P-value threshold of 10^-6 ^and lower, this supercluster was split into five subclusters, corresponding to COG565, COG0219, and three subfamilies of COG0566 we termed A, B, and C (Figure 5). In COG0566A we also included a group of apparent outliers which formed loosely connected subclusters at the periphery of COG0556.

**Figure 6:**
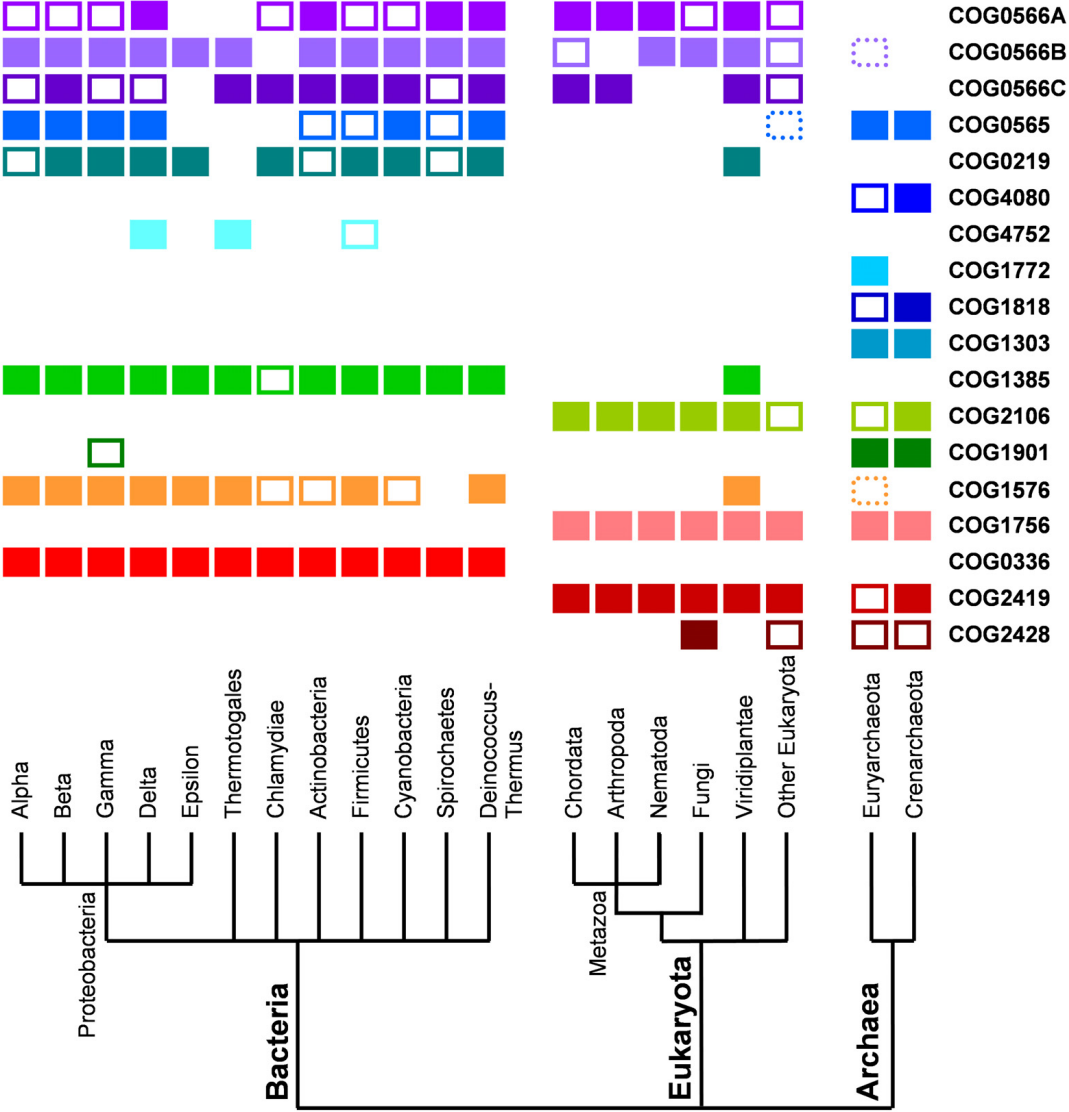
**Phyletic patterns of SPOUT families analyzed in this work, analyzed with respect to the fully sequenced genomes of organisms from the tree Domains of Life**. Full squares correspond to taxons, where at least 50% of fully sequenced genomes contained the member of a given COG. Note that taxons comprise different numbers of representatives, e.g. Thermotogales contain only one species, *Thermotoga maritima*. For taxons with more than one fully sequenced genome, the presence of a COG member in less than 50% of genomes is indicated by an empty square, and the presence of just a single member is indicated by a dotted square.

The final, refined set of all SPOUT members comprised 1629 sequences, grouped into 18 families (COGs) shown in Table 2. The phylogenetic distribution of their members has been analyzed using an in-house Python script and is shown in Figure 6. Interestingly, no single family is found to span all taxons, in all three Domains of Life.

**Table 2:** Distinctive Characters Describing Structural, Sequential, and Functional (Dis)similarities of SPOUT families

family	representative	specificity	1	2	3	4	5	6	7	8	9	10	11	12
COG0566A	TrmH	tRNA:Gm18	1	1	1	1	1	1	1	-	1	1	-	1
COG0566B	RlmB	23S rRNA:Gm2251	1	1	1	1	1	1	1	-	1	1	-	1
COG0566C	AviRb	23S rRNA:Um2479	1	1	1	1	1	1	1	-	1	1	-	1
COG0565	TrmJ	tRNA:Xm32	?	?	?	1	1	1	1	-	1	1	-	1
COG0219	YibK	unknown	1	1	1	1	1	?	1	-	1	1	-	1
COG4080	PAE3113	unknown	?	?	?	1	1	?	-	-	-	-	-	1
COG4752	TM1570	unknown	?	?	?	1	1	?	1	-	-	1	-	1
COG1772	PF0315	unknown	?	?	?	1	5	?	1	-	-	-	-	1
COG1818	PF1925	unknown	?	?	?	3	1	?	1	-	-	-	-	1
COG1303	PAB1040	tRNA:Cm56	?	?	?	1	1	1	1	-	1	-	-	1
COG1385	RsmE	16S rRNA:m^3^U1498	2	1	1	2	2	3	-	-	1	-	-	1
COG2106	MT1	unknown	3	1	1	4	6	?	1	-	-	1	-	1
COG1901	PF1403	unknown	?	?	?	2	2	?	-	-	-	-	-	1
COG1576	YbeA	unknown	5	2	2	5	3	?	-	1	-	-	-	0
COG1756	Nep1p	unknown	?	?	?	6	7	?	-	-	-	-	-	1
COG0336	TrmD	tRNA:m^1^G37	4	2	2	7	3	2	-	1	-	-	-	0
COG2419	Trm10p	tRNA:m^1^G9	?	?	?	8	4	2	-	1	-	-	1	?
COG2428	Yor021c	unknown	?	?	?	9	4	?	-	-	-	-	1	?

**1. DALI Z-score > 15 in pairwise similarities: **this feature clearly distinguishes four members of the 'supercluster' (COG0566 and COG0219, state 1) from other SPOUT structures (states 2–5). **2. DALI Z-score > 11 in pairwise similarities: **this feature allows to split the SPOUT structures into two groups: COG0566, COG0219, COG2106, and COG1385 (state 1) vs COG0336 and COG1576 (state 2). **3. Dimerization mode (perpendicular vs antiparallel): **this feature disregards the similarity of monomers, but focuses on their mutual orientation. As discussed earlier (see Figure 2), members of COG0566, COG0219, COG2106, and COG1385 form 'perpendicular' dimers (state 1), while members of COG0336 and COG1576 form 'antiparallel' dimers (state 2). **4. HHpred P-value < 1e-4 in pairwise similarities: **this feature assigns state 1 to COG0556, COG0219, COG0565, COG4080, COG1303, COG4752, and COG1772, state 2 to a pair of COG1385 and COG1901, and states 3–9 to all other COGs, regarded as 'singletons'. **5. Clustering according to CLANS: **here, states were arbitrarily assigned according to the visual analysis of CLANS results (Figure 4). This grouping identifies nearly the same group of closely related families as feature 4 (with the exception of inclusion of COG1818 and removal of COG1772), but allows additions groupings, in particular COG0336 with COG1576 (state 3) and COG2419 with COG2428 (state 4). Singletons are assigned states 5–7. **6. Methylation product: **assigned based on experimental data. State 1: 2'-O-ribose, state 2: m^1^G, state 3: m^3^U. **7. Active site variant I: **conserved Arg or Lys in the N-terminal helix and conserved Ser or Thr in the C-terminal helix. In this manuscript we predict that these residues may be characteristic for 2'-O-ribose MTases. **8. Active site variant II: **conserved Arg or Lys in the C-terminal helix. This feature may be correlated with the antiparallel dimerization mode (feature 3), which requires 'migration' of the basic residue in one subunit of the dimer (compared to the 'perpendicular' mode of dimerization) to maintain the interaction with the rest of the active site in the other subunit. **9. Conserved Glu in the cofactor/methionine-binding loop: **the functional significance of this conservation is unclear; this residue forms an intramolecular salt bridge and seems more important for the structural stability than for catalysis. **10. Insertion (> = 4aa) in a loop that binds adenosine of AdoMet. 11. Insertion (> = 4aa) in a loop that binds methionine of AdoMet. 12. Secondary structure of the variable Rossmanoidal part of the SPOUT fold: **state 1:β-strands, state 0: 2 β-strands.

**Figure 7:**
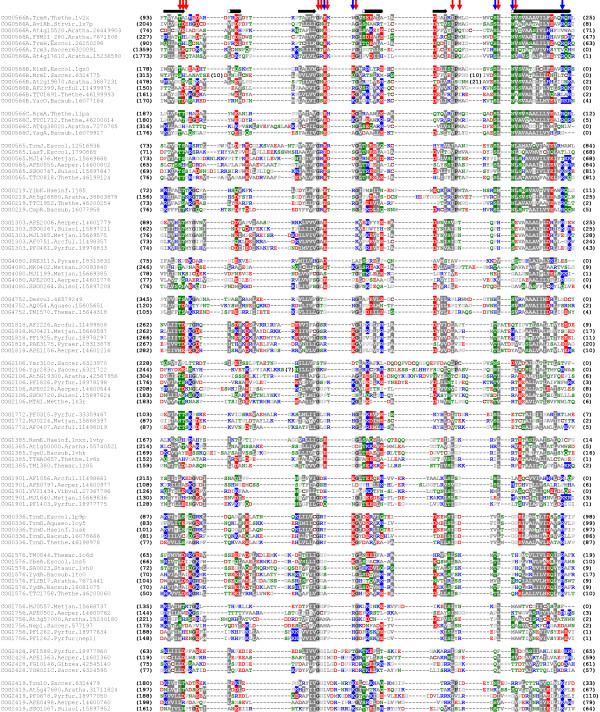
**Multiple sequence alignment of selected representatives of SPOUT COGs**. Sequences are denoted by the COG number, species' name, the NCBI gene identification (GI) number and the PDB code (if applicable). The variable termini and non-conserved insertions are not shown; the number of omitted residues is indicated in parentheses. Amino acids are colored according to the physico-chemical properties of their side-chains (negatively charged: red, positively charged: blue, polar: green, hydrophobic: grey). The consensus secondary structure is shown above the alignment as tubes (helices) and arrows (strands). The most typical positions of AdoMet-binding residues are indicated above the alignment by vertical red arrows, while the typical positions of catalytic residues are indicated by blue arrows. Note that additional catalytic residues may be present in the N-terminal part (unalignable and therefore not shown in this figure) and that the position of catalytic residues varies between families, e.g. it depends on the mode of dimerization.

Based on the superposition of structurally characterized SPOUT members, pairwise family-family alignments obtained using HHSearch and sequence-structure alignments produced by FR methods (see above) we generated a multiple sequence alignment of representatives of all SPOUT COGs (Figure 7). It illustrates the weak conservation of amino acid residues in the structurally conserved AdoMet-binding site and very strong variability of other regions between most families. This analysis shows that SPOUT MTases do not share a universally conserved active site, and suggests that different families developed specialized versions of catalytic pockets to carry out methylations of their substrates. It is therefore important to find out if different families that exhibit similar activities on different substrates (e.g. methylation of ribose in different RNAs or N1-methylation of guanosine in positions 37 and 9 of tRNA) possess similar or different active sites.

### The central 'supercluster' groups together ribose 2'-O-MTases

The central supercluster (Figure 5) is formed by COG0219, COG0565, and COG0566 families. COG0219 groups together bacterial proteins represented by the putative MTase YibK from *Haemophilus influenzae *(1mxi in the Protein Data Bank [29]). Members of the YibK family contain only the SPOUT domain. COG0565 comprises bacterial and archaeal proteins exemplified here by the uncharacterized protein LasT and the recently characterized tRNA:Xm32 MTase (TrmJ) from *E. coli *[30]. All members of COG0565 contain the SPOUT domain with a C-terminal helical extension of unknown function, which may form an independently folded domain [30]. Surprisingly, a few functionally uncharacterized members of COG0565 contain also putative deoxyribonuclease domains: ELI_05635 from *E. litoralis *has an N-terminally fused GIY-YIG domain [23], while a small group of proteins from Alphaproteobacteria exemplified by bll5746 from *B. japonicum *have a C-terminally fused PD-(D/E)XK domain [31]. Orthologs of this PD-(D/E)XK domain (DUF559) in other Alphaproteobacteria are standalone open reading frames. It will be interesting to determine the function of these nuclease domains and find out if their activity is linked with the MTase activity of the SPOUT domain.

As mentioned above, the largest COG0566 was split into three subfamilies termed A, B, and C. All three subfamilies contain eukaryotic and bacterial proteins, and only one member from Archaea. COG0566A comprises orthologs of tRNA:Gm18 MTase (TrmH, EC 2.1.1.34) and is represented by a protein from *Thermus thermophilus *(1v2x in PDB [9]). Prokaryotic members of this subfamily contain only the catalytic domain, with short helical extensions at both termini, which do not form an independent domain. Eukaryotic members of COG0566A, including Trm3p from *S. cerevisiae*, which exhibit the same specificity as TrmH [8], lack the short helical extensions, but contain very long (often > 1000 aa) N-terminal domain of unknown function, predicted to contain multiple α-helices that may form a large superhelix. COG0566B comprises orthologs of 23S rRNA:Gm2251 MTase RlmB from *E. coli *(1gz0 [3]) and a paralogous lineage, comprising products of an apparent duplication within Gammaproteobacteria, exemplified by a hypothetical ribose MTase YfiF from *E. coli *[32]. Eukaryotic members are represented by the TAR (HIV) RNA binding protein 1 (TRBP1) in humans (2ha8 in PDB), and its ortholog Mrm1p (formerly Pet56p) in *S. cerevisiae*, which introduces a highly conserved Gm2270 modification in mitochondrial 21S rRNA (corresponding to Gm2251 in bacterial 23S rRNA) and has an essential role in the maturation of the mitochondrial large ribosomal subunit that is independent of its MTase activity [33]. This additional essential role of Mrm1p is not shared by RlmB, which is dispensable in *E. coli *[34]. COG0566C comprises close homologs of 23S rRNA:Um2479 MTase AviRb from *Streptomyces viridochromogenes *(1x7o in PDB [35]), including the functionally uncharacterized, putative 2'-O-ribose MTase RrmA from *T. thermophilus *(1ipa [2]), and the human protein RNMTL1. In both COG0566B and COG0566C the SPOUT domain is N-terminally fused to the L30e-like domain, which is probably involved in RNA binding. Interestingly, eukaryotic members of COG0566B, many eukaryotic members of COG0566C, as well as YfiF and its orthologs from COG0556B, but not other members of these families, contain an N-terminal positively charged (presumably disordered) N-terminal extension.

Our classification of proteins from the central supercluster partially differs from that proposed by Anantharaman et al. in the original article that defined the SPOUT superfamily [1]. The previously defined "SpoU family 1" overlaps not only with all three subfamilies of COG0566 proposed here, but also with COG0219 and a part of COG565, while the other part of COG0565 was suggested to be a separate "SpoU family 2". Our results clearly suggest that proteins such as MJ1476 (GI:15669668) belong to a separate family together with TrmJ (formerly YfhQ) and LasT from *E. coli *(COG0565) rather than with members of COG0566, as originally proposed.

Superposition of crystal structures and analysis of the resulting sequence alignment (Figure 7) reveals that all members of COG0219, COG0565, and COG0566 families share a common set of residues implicated in catalysis, delivered by both subunits in a dimer (e.g. R41 and S150 in TrmH) [14,35], which suggests that they are all active as RNA 2'-O-ribose MTases. We have experimentally verified this prediction for the YfhQ protein from *E. coli*, which was found to exhibit the tRNA:Xm32 MTase (TrmJ) activity [30].

### Other genuine and potential RNA 2'-O-ribose MTases

In the CLANS analysis of SPOUT protein (Figure 4), the core group is surrounded by four clusters: COG04752, COG4080, COG1772, and COG1303. COG4752 is a small family comprising a few bacterial proteins with erratic phyletic distribution, mostly from extremophiles (e.g. *Thermotoga maritima*, *Synthropus acidotrophicus*), but also from pathogens such as *Fusibacterium nucleatum *COG4752 was dubbed 'SpoU family 5' by Anantharaman et al. [1], implying a functional link with 2'-O-ribose MTases. On the other hand, COG4080 and COG1772 are identified as SPOUT superfamily members and predicted as MTases only in this work. COG4080 is a small family of archaeal proteins, it consists only of the SPOUT domain. As mentioned earlier, these proteins have been incorrectly classified in sequence databases as homologous to RecB. COG1772 is a small family of functionally uncharacterized proteins mostly from hyperthermophilic Euryarchaeota, represented here by PF0315 from *Pyrococcus furiosus*. COG1303 is the last 'satellite' of the supercluster. This exclusively archaeal family is represented here by the PAB1040 (GI: 14521775/5458995) protein from *Pyrococcus abyssi*, which was recently found to introduce the Archaea-specific 2'-O-metylation of C56 in tRNA [36].

Analysis of sequence conservation in COG4752, COG4080, COG1772, and COG1303 families (Figure 7) with the aid of comparative models of the representative proteins (data not shown) reveals that COG4752 and COG1303 exhibit a conserved Arg residue at the same position as the catalytic Arg of the orthodox 'supercluster' members. COG1772 members possess conserved Arg residues in the same helix (R36 and R32 in the representative sequence PF0315). Their shift by one and two turns (3 and 7 residues) towards the N-terminus with respect to the position equivalent to R41 of TrmH could be either genuine or an alignment/modeling artifact; nonetheless, it can be reliably predicted that the long side chain(s) of the conserved Arg residue(s) in COG1772 could assume a similar spatial position to the catalytic Arg of bona fide ribose MTases. Members of COG1772 also possess a conserved Ser (S166 in PF0315) at a position homologous to the catalytic Ser of the 'supercluster', while members of COG4752 possess a conserved Ser at the position of conserved Ser in COG1303 (i.e. 2 residues towards the C-terminus compared to members of the 'supercluster'). Altogether, this suggests that the putative active sites of COG1772 and COG4752 are similar to those of genuine 2'-O-ribose MTases (COG0565/0566/1303) and therefore these proteins are likely to exhibit a similar activity. On the other hand, members of COG4080 do not seem to possess any conserved Arg or Ser residues at positions equivalent to those characteristic for 2'-O-ribose MTases, which suggests that despite their close relationship to COG0565/0566/0219, they may exhibit a different function.

Among other SPOUT families, only the eukaryo-archaeal COG1818, dubbed "SpoU family 4" by Anantharaman et al. [1], exhibits conserved Arg and Thr residues (R197 and T328 in PF1925, common to bona fide 2'-O-ribose MTases (Figure 7). Therefore, we predict these proteins are also involved in ribose methylation.

COG2106 appears to be an outlier. It groups together functionally uncharacterized proteins from Archaea and Eukaryota, of which one (functionally uncharacterized putative MTase MT1 from *Methanothermobacter thermoautotrophicus*) had a structure determined by the structural genomics factory (1k3r in PDB, [37]). A characteristic feature of this family is an insertion of the OB-fold domain [19] into the SPOUT domain. MT1 dimerizes in a perpendicular fashion, similar to 2'-O-MTases. It also exhibits a conserved Arg common to the catalytic Arg of 2'-O-MTases (R33 in MT1, replaced with Lys only in a few eukaryotic members of COG2106), and has a conserved (but not invariant) Thr residue (T243) at the position of the catalytic S150 of TrmH. Thus, methylation of ribose appears to be a possible function of MT1 and other members of COG2106, but at this point other activities cannot be excluded (and in fact, phylogenetic considerations suggest that COG2106 may be orthologous to bacterial m^3^U MTases from COG1385, see below). Interestingly, COG2106 has two members in *S. cerevisiae*, Ygr283cp and Ymr310cp, of which the latter appears to be more diverged (Figure 7). It remains to be verified experimentally, if these two yeast proteins are independent enzymes with similar functions but perhaps different specificities, or if they form a heterodimer in which each subunit fulfills a slightly different function, as has been found for other yeast RNA modification and processing enzymes, including tRNA:m^1^A58 MTase Trm6p/Trm62p [38], tRNA deaminase Tad2p/Tad3p that converts A34 to inosine [39], or a heterotetrameric intron-splicing nuclease Sen [40].

### Matching of predicted 2'-O-ribose MTases and known ribose methylation sites

The remaining predicted but still unproven 2'-O-ribose MTases from the SPOUT superfamily in *E. coli *include YibK, LasT, and YfiF, which all belong to the 'supercluster'. The fourth predicted 2'-O-ribose MTase in *E. coli *is encoded by the *ygdE *gene, and belongs to the unrelated RFM superfamily [41]. On the other hand, there are four known sites of ribose methylation in *E. coli *RNA, for which no enzyme/ORF has been assigned yet: a monomethylated residue Cm at position 2498 in 23S rRNA, a dimethylated residue m^4^Cm at position 1402 in 16S rRNA, Cm at position 34 in tRNA^Leu5^, and a hypermodified nucleoside 5-carboxymethylaminomethyl-2-O-methyluridine (cmnm^5^Um) at position 34 in tRNA^Leu4 ^[42,43]. It remains to be determined experimentally, if these four predicted 2'-O-ribose MTases are responsible for the last four unassigned ribose methylations. In our hands, YibK and LasT have no activity on the position 34 in tRNA^Leu5 ^[30], which further narrows down the prediction of this activity for YgdE or YfiF. Interestingly, the *yfiF *gene has been independently analyzed by de Crecy-Lagard and predicted to encode a tRNA:m^2^A37 MTase (TrmG) [44] based on the similarity of its genomic location (58 min of the *E. coli *chromosome) with the location experimentally determined for the *trmG *gene (56–61 min) [45]. While we cannot exclude the possibility that YfiF is involved in m^2^A methylation, we would like to note that methylation of an endocyclic carbon atom (C2 in m^2^A) is chemically more difficult than methylation of ribose or of an exocyclic amino group, and may require a different mechanism, such as formation of a covalent protein-nucleic acid intermediate that has been observed in m^5^U or m^5^C MTases acting on RNA or DNA [46]. YfiF and its orthologs, however, exhibit conservation of all residues characteristic for 2'O-MTases, and their presumptive active site does not show any similarity to known MTases acting on endocyclic carbon atoms. We hope our analysis will stimulate experimental analyses of YfiF that will solve this controversy and provide important piece of data for the ultimate identification of the gene encoding the TrmG enzyme.

Prediction of specificities for potential ribose MTases from organisms other than *E. coli *is unfortunately very difficult – first, because the total number and positions of all modifications in all RNAs have not been determined for any other organism and second, because Eukaryota and Archaea use an additional system of ribose 2'-O-metylation, which is based on a single enzymatic complex, whose specificity is governed by C/D box small RNAs [47]. These guide RNAs are not trivial to detect, their complete catalog is not known for any cell [48], and it has been demonstrated that they may be involved in modification of some nucleosides that in the same or in a very closely related organism can be also modified by a standard mechanism, involving a site-specific RNA MTase [36,49]. Thus, the determination of activities of predicted ribose 2'-O-MTases that lack orthologs in *E. coli *will require complementary efforts to determine the number and position of modifications in the substrate RNAs. In Archaea and Eukaryota this effort should be also coordinated with identification and characterization of C/D box small RNAs.

### m^3^U MTases and their relatives

COG1385 is an almost exclusively bacterial family (with a few members in plants) represented here by the recently characterized RsmE enzyme from *Escherichia coli *that carries out N3-methylation of U1498 in 16S rRNA [12]. Crystal structures of COG1385 members RsmE from *Haemophilus influenzae *(1vhy in PDB [50]), YqeU from *Bacillus subtilis *(1vhk in PDB [51]), Tt1573 from *T. thermophilus *(1v6z in PDB) and Tm1380 from *Thermotoga maritima *(1z85 in PDB) have been determined, demonstrating the presence of the characteristic knot, yet without providing hints as to the details of the molecular function. All structurally characterized members of COG1385 exhibit the 'extended' version of the core β-sheet (topology 6↑-4↑-5↑-1↑-2↑-3↑) and dimerize in a 'perpendicular' mode similar to 2'-O-MTases. Their characteristic feature is the N-terminal PUA domain implicated in RNA-binding [20]. Analysis of sequence conservation in the light of the crystal structures (data not shown) reveals that the putative active site of RsmE is again located at the subunit interface (R224 in monomer A, and Q141 and perhaps also E101 in monomer B). R224 is homologous to N152 of TrmH (Figure 7), while the 'swapped' residues E101 and Q141 are located in the first two N-terminal helices of the SPOUT domain, i.e. differently than the 'swapped' residues of either 2'-O-ribose MTases or the TrmD family that are located in the C-terminal helix. According to sequence comparisons by HHsearch and CLANS, COG1385 appears to be related to the uncharacterized family of proteins present in Archaea, grouped together in COG1901. However, these two families do not seem to share conserved residues that could be involved in catalysis and therefore we cannot reliably predict the function of COG1901 members.

### m^1^G MTases and their relatives

COG0336 comprises exclusively bacterial orthologs of the tRNA:m^1^G37 MTase TrmD, here represented by the protein from *E. coli *(1p9p in the PDB [11]). TrmD is one of the few members of the SPOUT superfamily that have been relatively well characterized by site-directed mutagenesis, and its active site is quite well defined [10,11] even though the structure of the enzyme-substrate complex still remains to be determined. The characteristic features of TrmD are: the "minimal" version of the core β-sheet (topology 7↑-5↑-6↑-1↑-2↑), with the Rossmanoidal domain composed only of two α/β segments, an additional β-hairpin (1↑-2↓), and a small helical C-terminal domain, which is swapped between the two monomers in the dimer (Figure 1). As mentioned earlier, TrmD dimers are formed in an antiparallel way (Figure 2). The active site of TrmD is also located at the subunit interface and comprises residues from two monomers: E116, R154, and D169.

Of all SPOUT members, only COG1576 exhibits some obvious similarities to TrmD. This family groups together mostly bacterial proteins. None of its members have been characterized functionally. However, four crystal structures have been determined by two structural genomics consortia: for YbeA from *E. coli *(1ns5), YydA from *Bacillus subtilis *(1to0), SAV0024/SA0023 from *S. aureus *(1vh0, [51]), and Tm0844 from *T. maritima *(1o6d, [51]). Remarkably, all these proteins share the "minimal" version of the core β-sheet (topology 5↑-3↑-4↑-1↑-2↑) and the antiparallel dimerization model with the TrmD family. However, members of COG1576 lack the additional β-hairpin and the C-terminal domain of TrmD, and therefore they can be regarded as 'minimal' (in the structural sense) members of the SPOUT superfamily. The available crystal structures of COG1576 members exhibit also the smallest dimerization interface, so small that according to PDB and the Protein Quaternary Structure Database (PQS) 1o6d has been classified as a monomer, even though in the crystal it forms protein-protein contacts with its symmetry mate (-X -Y, Z) that are very similar to those found in 1ns5, 1to0, and 1vh0 dimers (data not shown). Members of COG1576 shares a common conserved Arg residue (R142) with TrmD, however their putative active site comprises conserved residues that are not present in TrmD, including T126, Y152, and H153. Since the COG1576 family includes a member from *E. coli *(YbeA), where all m^1^G modifications in tRNA and rRNA have been linked to the respective enzymes, it is unlikely that these proteins carry out *N*^1^-methylation of guanosine. However, they may be involved in generation of a chemically similar product with a methylated endocyclic nitrogen atom, e.g. m^3^Ψ1915 in 23S rRNA.

Sequence analysis of COG2419 (represented by the tRNA:m^1^G9 MTase Trm10 [52]) reveals virtually no common conserved residues with the functionally similar tRNA:m^1^G37 MTase TrmD (Figure 7). This implies that m^1^G methylation either appeared independently at least two times in the history of SPOUT MTases or that considerable sequence divergence has led to a complete redesign of MTase-guanosine interactions since the putative ancestral m^1^G MTase that has given rise to the TrmD (COG0336) and Trm10p (COG2419) families. The independent origin of the same catalytic activity has been already reported for unrelated enzymes from the Rossmann-fold MTase (RFM) superfamily, namely the eubacterial RlmA family specific for N1-methylation of G745 or G748 in 23S rRNA [53,54] and archaeo-eukaryotic Trm5 family specific for G37 in tRNA (i.e. analogous to TrmD in bacteria [55]), which possess very different active sites for the same reaction (our unpublished data). Interestingly, COG2419 does not seem to share any strongly conserved residues even with its closest relative COG2428 (which was predicted as a family of putative MTases by Ginalski et al. [18], without providing any hints as to the identity of the active site). Our protein fold-recognition analysis of COG2419 and COG2428 members confirmed their membership in the SPOUT superfamily. However, target-template alignments returned by different fold-recognition methods varied greatly and therefore the structural predictions and the resulting alignments with other SPOUT members are the least confident among all sequences analyzed in this work. Thus, we would like to propose COG2419 and COG2428 members as attractive targets for structure determination by X-ray crystallography and for detailed analysis of the architecture of the active site and the biochemical mechanism of action.

### Proteins involved in ribosome biogenesis are predicted novel SPOUT MTases

COG1756 is a novel addition to the SPOUT superfamily. It groups together proteins from Archaea and Eukaryota. It is represented by the Nep1p protein from *S. cerevisiae*, which has been implicated in pre-rRNA processing and its indirect role in methylation during ribosome biogenesis was supposed [56]. Recently, genetic evidence have been presented for the role of Nep1p in 18S rRNA binding and an Rps19p assembly [57]. Interestingly, it was found that Nep1p binds RNA sequences that share a six-nucleotide consensus motif C/UUCAAC, including the sequence of nucleotides 1553–1577 in the 18S rRNA. This region encompasses two modified nucleosides: m^7^G1573 modified by the yet unknown enzyme and Gm1570 modified by the fibrillarin complex guided by the C/D box snoRNA snR57. The deletion of snR57 RNA and multicopy expression of the ribosomal 40S subunit protein Rps19p can partially suppress the growth defect caused by the deletion of Nep1p-encoding gene. Thus, it was suggested that Nep1p binds to helix 47 of the 18S rRNA and possibly supports the association of Rps19p to pre-ribosomal particles [57]. Direct involvement of Nep1p in methylation of G1570 was considered unlikely, because deletion of snR57 in yeast leads to a complete absence of the Gm1570 modification despite the presence of Nep1p. On the other hand, Nep1p ortholog is present in *Sulfolobus solfataricus*, which lacks m^7^G1573, thus arguing against the involvement of Nep1p also in G1573 modification [57].

The identification of the SPOUT domain in COG1756 suggests that Nep1p may be enzymatically active as an AdoMet-dependent MTase, whose action can somehow interfere with the snoRNA-guided methylation of G1570. Analysis of sequence conservation in COG1756 reveals invariant and early invariant residues in the structurally variable N-terminus that cannot be modeled with confidence rather than in the structurally conserved knotted C-terminus. Therefore, the architecture of the potential active site cannot be predicted with confidence and only a number of potentially functionally important residues can be proposed for experimental analysis: E51, R88, D90, S102, R129. At this stage it is not possible to confidently predict the biochemical function of Nep1, however, site-directed mutagenesis of conserved residues might discriminate between the catalytic and possible non-catalytic roles of Nep1p in ribosome biogenesis (e.g. 'chaperoning' of the ribosome assembly by interacting with RNA and ribosomal proteins) and reveal its molecular mechanism of action.

### Structural and evolutionary classification of the SPOUT superfamily

**Figure 8:**
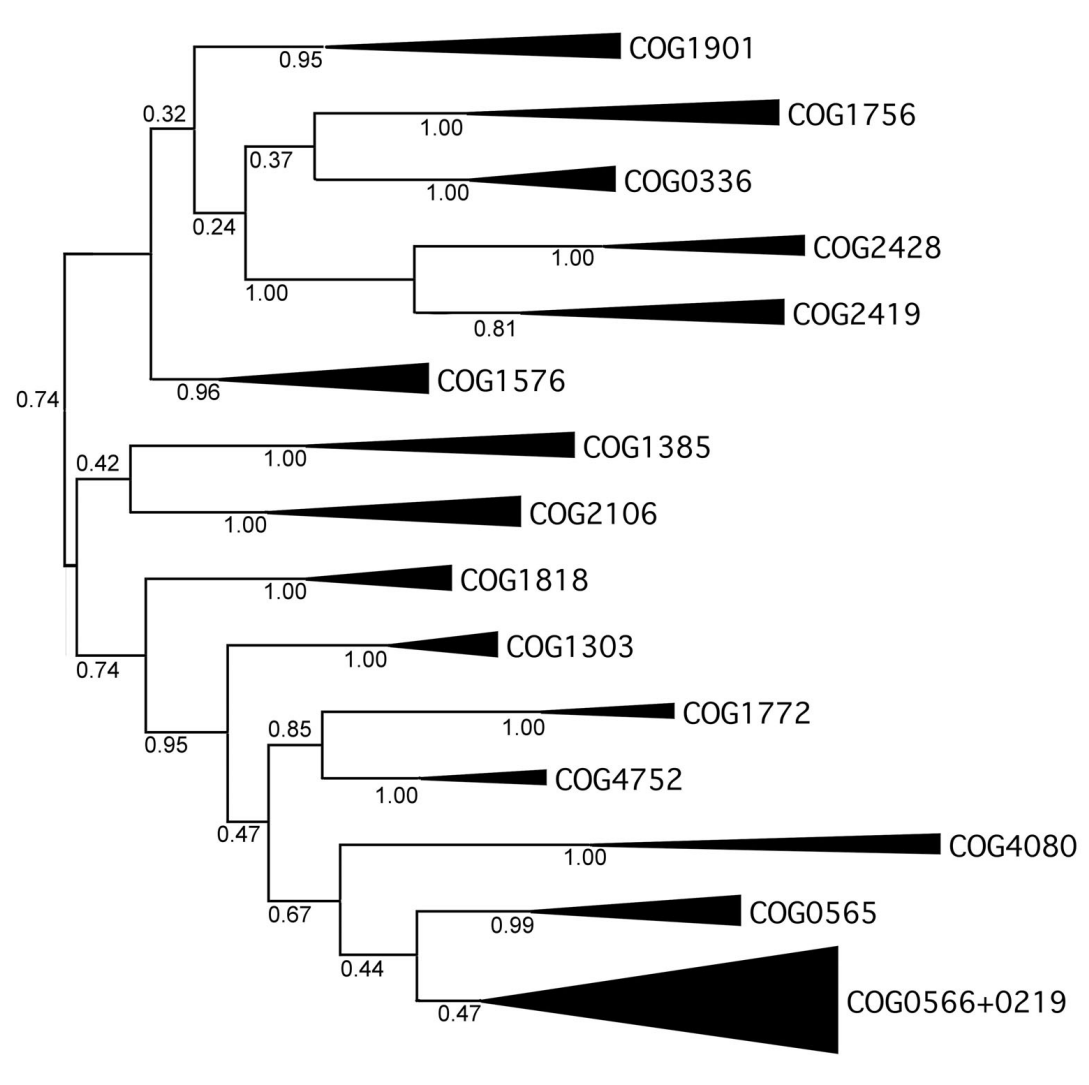
**Conventional sequence-based Bayesian tree of SPOUT MTases calculated based on the sequence alignment in Figure 7**. Triangles labeled with COG names indicate monophyletic families. All branches are labeled with their posterior probabilities. Although the monophyly of all individual COGs and some groups of COGs is well supported, the supports of deep branches is poor (<0.5).

Based on the sequence alignment of the structurally conserved (and therefore reliably alignable) knot region of SPOUT MTases (Figure 7) we attempted to infer the phylogenetic tree of the superfamily. However, traditional methods for phylogenetic reconstruction based on sequence data, including the neighbor-joining (NJ), maximum parsimony (MP), and maximum likelihood (ML) failed to produce a confident tree with well-resolved branches. The NJ and MP methods failed apparently due to the high sequence divergence and uneven rates of evolution between and within different lineages, while for the ML tree the bootstrap support could not be calculated due to prohibitively long computation time. Thus, we decided to use the Bayesian approach, which combines the relative reliability of ML with the fast scanning of the parameter landscape by the Markov chain Monte Carlo (MCMC) approach implemented in the program MrBayes3 [58]. Figure 8 shows a Bayesian tree of SPOUT sequences, which suggests that known ribose 2'-O-MTases together with m^3^U MTases, to the exclusion of m^1^G MTases, while. The branches, however, received low support, and the topology was sensitive to the choice of parameters (e.g. substitution matrices, models of substitution etc.)

Because protein structure is much more conserved than protein sequence, it allows making evolutionary inferences through comparative structure analysis – either without any consideration of sequence [59] or by combination of sequence and structure data. Initially, we attempted to infer a tree for representative SPOUT structures using several methods for calculation of 'structural' distances via the STRUCLA server [60]. However, the resulting trees were even less robust than those based solely on sequence and no meaningful consensus could be derived (data not shown). Thus, we sought to determine the phylogenetic tree of the SPOUT superfamily using an approach similar to the one recently used to resolve relationships in the protein kinase-like superfamily [61], namely a unified Bayesian analysis that incorporates the sequence alignment and other features, including structure data, discretized into 'characters'. This approach often yields improved resolution of both close relationships (owing to the criterion of sequence divergence, with nearly identical structures) and remote ones (owing to the criterion of structural divergence, with sequences diverged to the level of random noise). It is noteworthy that in the case of the SPOUT superfamily this approach allowed us to extend the quantitative comparison to sequence and structural features that span sequence regions outside the common core corresponding to the knotted region (Figure 7). The N-terminal part of the SPOUT domain is extremely diverged and contains variable number of secondary structure elements, making this region spatially non-superimposable for some members, and virtually precluding the creation of a full-length alignment, in particular for those families, which lack structurally characterized representatives. Nonetheless, different SPOUT subfamilies do exhibit clear pairwise similarities or share various common features also in the N-terminal region, and these data can be incorporated in the unified Bayesian analysis along with the sequence alignment data for the knotted C-terminal part.

We decided to complement the alignment data with several different types of characters, with their states given in a numbered code (Table 2). In features 1–3 we considered global structural similarity between the experimentally determined structures, which is a very strong direct indicator of evolutionary relationship. In particular features 1 and 2 relied on comparison between the isolated, monomeric SPOUT catalytic domains using DALI [16] (see Table 1). A particular 'state' was assigned to members of clusters identified according to the single linkage criterion with an arbitrary threshold of similarity. 'Singletons' were considered as different states. The state of features 1–3 requires experimentally determined structures, they cannot be reliably assessed for proteins of unknown structure (not even for comparative models), therefore for the majority of COGs they remained unassigned for the Bayesian analysis and are indicated as '?' in Table 1. In features 4–5 we considered global sequence similarity, which has the advantage of comparing all SPOUT families, regardless of the availability of structures. Moreover, as with the structural comparisons by DALI, it permits comparing and detecting similarity between the N-terminal segments. The assignment of states for features 4 and 5 was done in a similar way as for features 1 and 2. Feature 6 is the type of the methylated nucleoside generated in the reaction: obviously, for the functionally uncharacterized SPOUT families the state of this feature is unassigned. Features 7–11 identify conserved residues or structural peculiarities – here 1 indicates that the character is present, while its absence is regarded as an unassigned state '-' (the missing feature is regarded identically as '?' i.e. variants with a particular feature are grouped together, but those without it may or may not to be grouped together). Feature 12 concerns the presence or absence of a highly variable strand in the N-terminal region, which can be determined from the structures or inferred from secondary structure predictions. Here, 1 indicates that the character is present (the N-terminal variable region comprises 3 strands), 0 that it is absent (the N-terminal variable region comprises 2 strands), while for two families the state could not be determined ('?') due to the absence of experimentally determined structure and highly uncertain secondary structure prediction

**Figure 9:**
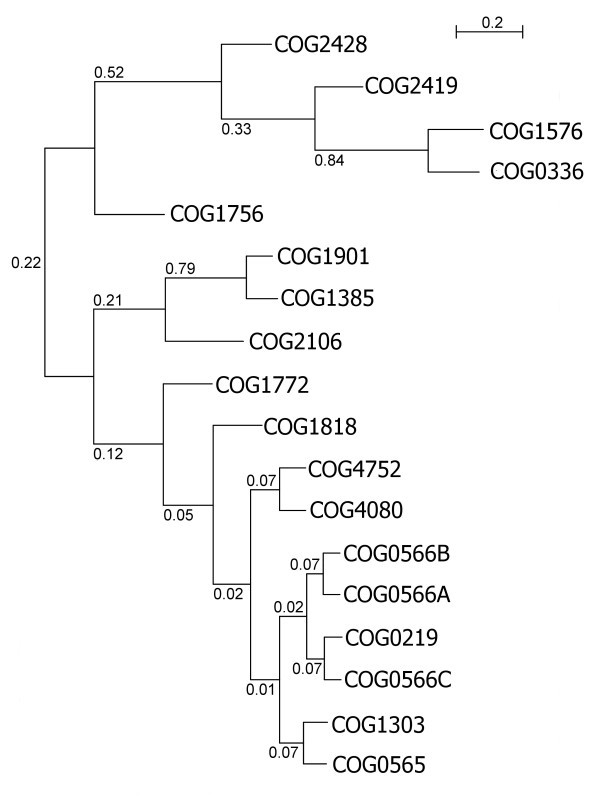
**Bayesian tree of SPOUT COGs based on the feature character matrix in Table 2**. All branches are labeled with their posterior probabilities. Although the overall topology of the tree is similar to that of the sequence-based tree, features provide significant support only for a few lineages.

The phylogenetic value of characters from Table 2 was assessed by using them to calculate a Bayesian tree without any reference to the sequence alignment. The tree based on features (Figure 9) generally agrees with the tree based solely on amino acid distances (Figure 8): in particular, two main branches are apparent, one with m^1^G MTases and the other with known ribose 2'-O-MTases. However, only a few well-supported branches can be considered as informative, e.g. the grouping of four COGS: 0248, 2419, 0336, and 1576, and the grouping of COG1901 with COG1385. Other nodes of the feature-based tree are practically unresolved, including the entire branch of 2'-O-MTases. Interestingly, the position of a few 'uncharacterized' COGs differs between the sequence- and feature-based trees. In particular in the sequence-based tree COG1901 is found in the m^1^G branch with a moderate support, while in the feature-based tree is groups together with COG1385 (and in general with m^3^U and ribose MTases) with a strong support. Overall, the feature-based tree has better resolution for deep nodes (remote relationships) but does not distinguish between terminal nodes, while the sequence-based tree has reasonable resolution for terminal nodes, but does not provide confident estimation of remote relationships. Thus, both types of data show high potential for complementation.

**Figure 10:**
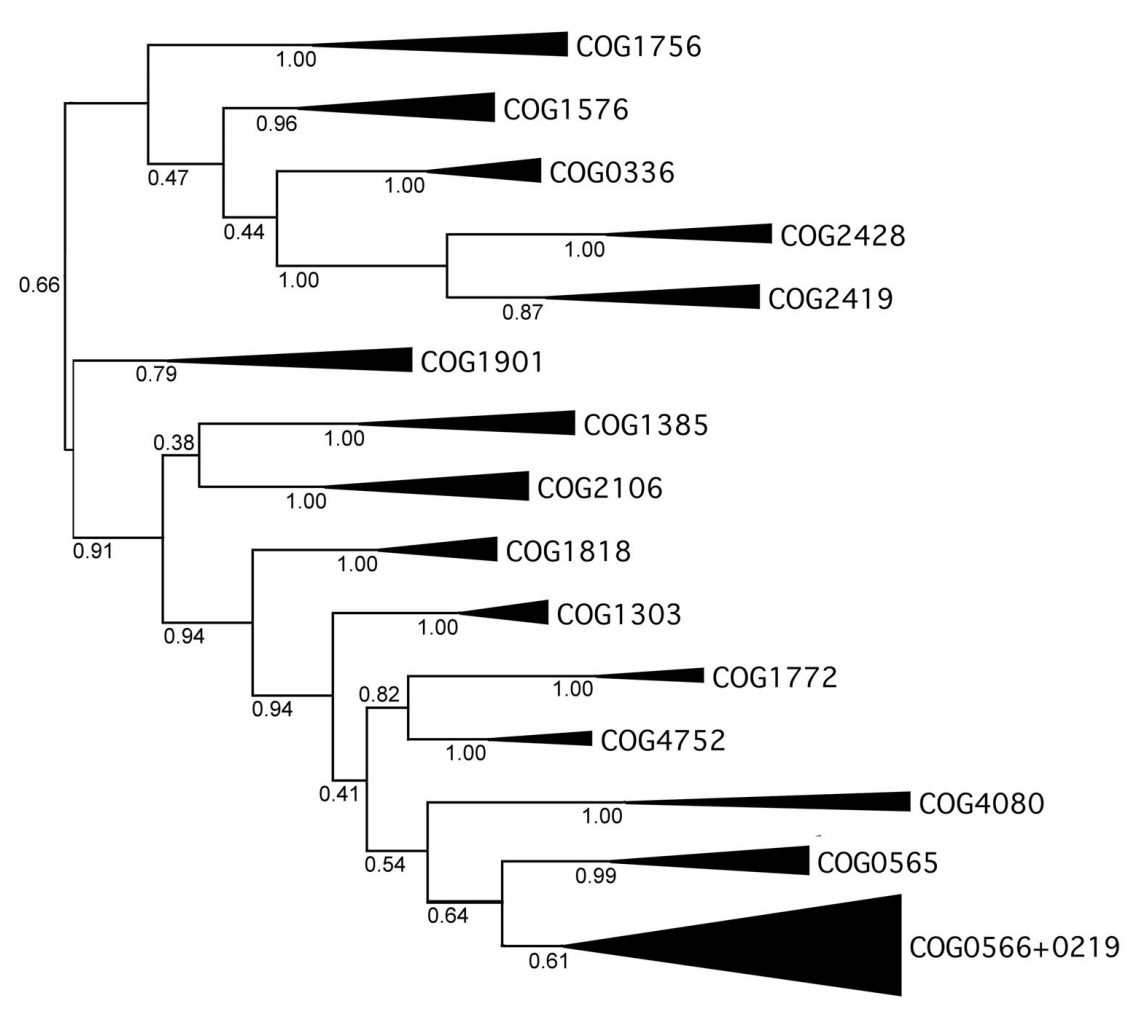
**The unified Bayesian tree of SPOUT MTases calculated based on the sequence alignment in Figure 7 and the character matrix in Table 2**. All branches are labeled with their posterior probabilities. Compared to the sequence-only tree, this tree shows improved support for deep branches and comparable support for terminal branches.

In order to meaningfully combine the alignment data with the feature data, we decided to increase the importance of structural information. Since the feature data comprise only 12 character types, we decided to 'strengthen' the feature data by multiplying the columns with 'structural' characters (features 1–3) by two. The resulting character matrix with 15 columns was incorporated into the input file to be quantitatively evaluated together with sequences as 'mixed' data, allowing the creation of a single Bayesian tree that provides maximum agreement with both sequences and features. The resulting tree (Figure 10) exhibits the most important features exhibited both by the sequences-only tree and by the features-only tree, in particular the presence of a well-resolved division between Xm and m^1^G MTases, but now both the deep and terminal nodes receive better support than in any of the original trees and the mutual relationship of many families can be resolved with confidence. Only the position of COG1901 is unresolved – the combination of sequence data for the knotted region pulling it towards m^1^G MTases and the data on overall features pulling it towards COG1385 resulted in artificial placement of COG1901 at the root. Moreover, the internal structure of the 'supercluster' (COGs 0219, 0565, 0566) branch remains unresolved (as It was both original trees), mostly due to the abundance of highly conserved residues in the aligned knotted region (e.g. paucity of informative sites that would help to distinguish between subfamilies) and virtual identity according to the structural and functional features (nearly identical structures, very similar functions).

**Figure 11:**
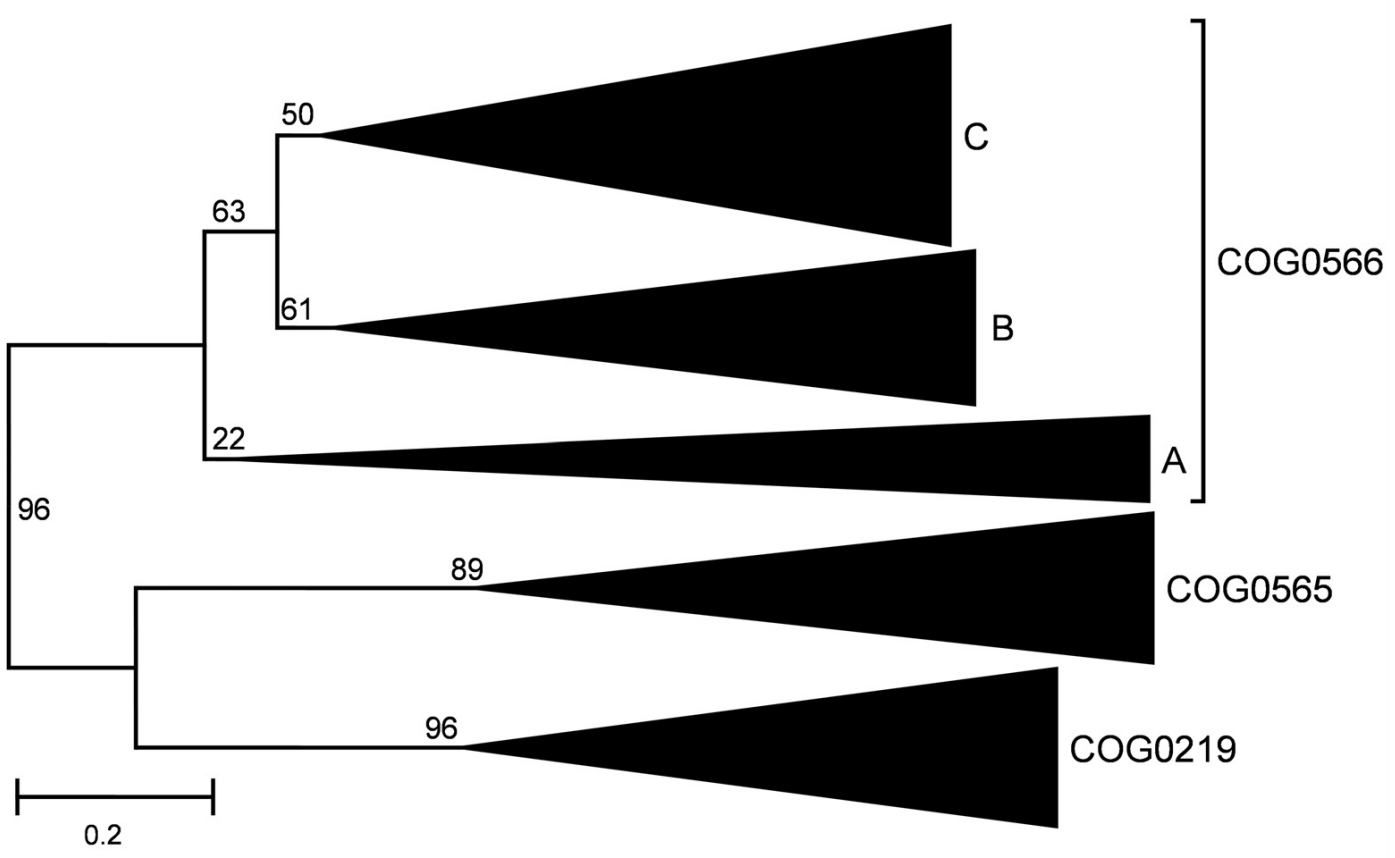
**Minimum evolution tree of the 'supercluster' (COG0219, COG0565, COG0566)**. Triangles labeled with COG names indicate monophyletic families. All branches are labeled with the support according to the interior branch test.

To obtain a meaningful tree for the closely related members of COGs 0219, 0565 and 0566 (A, B, and C) that could not be separated in the SPOUT tree, we constructed a multiple sequence alignment and calculated a Maximum Evolution tree (Figure 11, see Methods for details). All five families form monophyletic branches, in agreement with the classification based on the results of CLANS analyses. The monophyly of COG0219, COG0565, and the whole COG0566 is strongly supported. The classification of COG0566 into three families receives relatively low statistical support, indicating that divergence within each subfamily is comparable to the divergence between the subfamilies. Thus, duplications within COG0566 occurred probably shortly before the radiation of all major bacterial lineages. However, COG0566B and COG0566C group together to the exclusion of COG0566A, in agreement with their predicted common origin from one of the two first copies of the ancient COG566 MTase that acquired a L30e-like domain.

### Interpretation of the phylogenetic trees: origin and evolution of the SPOUT superfamily

A major question in evolutionary analyses of protein (super)families concerns the time of their origin and appearance of the main lineages with respect to the Last Universal Common Ancestor of all contemporary cellular organisms (LUCA). For the SPOUT superfamily, this has been subject to controversy. In the analysis of AdoMet-binding proteins reported by Mushegian, only the predecessor of COG0565 is thought to has been present in LUCA [5]. On the other hand, Ouzounis and coworkers predicted the members of both COG0566 and COG0565 to be present in LUCA; COG1385 and COG1576 were hypothesized to be present in LUCA as well, but only under a more relaxed model of gene loss [62]. However, these inferences have not taken into account the possibility that some COGs with (partially) complementary phylogenetic distributions may be orthologous. Here, we attempted to infer the evolutionary history of the whole SPOUT superfamily and predict its state in LUCA by interpreting the tree in the light of the phyletic distribution of different COGs (Figure 6).

Although a few SPOUT families possess members in all three Domains of Life, Bacteria, Archaea and Eukaryota, some of them might have expanded to the contemporary phylogenetic range not by vertical descent, but by the horizontal gene transfer (HTG). In particular, the single archaeal members of COGs that are almost exclusively bacterio-eukaryotic (COG0566: one archaeal representative in *Archaeoglobus fulgidus*) or bacterial (COG1576: one archaeal representative in *Methanococcus maripaludis*) have been almost certainly acquired by HGT from Bacteria living in similar environments. Conversely, a few bacterial members of a typically archaeal COG1901 could have been acquired by HGT from Archaea. Bacterio-archaeal COG0565 possesses a single eukaryotic member in *Entamoeba histolytica*, which was probably acquired by HGT from a bacterium. In addition, several almost exclusively bacterial COGs such as COG0219, COG1576, COG0336, and COG1385 possess eukaryotic members only in plants, which suggests that the latter have been acquired by HGT from the chloroplast endosymbiont and the respective families can be regarded as of bacterial origin. The sparsely populated bacterial COG4752 could have evolved by duplication and/or HGT from one of the bacterial families within the supercluster or from one the archaeal COGs – 'satellites' of the supercluster (Figure 4).

Some COGs that appear as neighbors in the tree, exhibit identical or very similar phyletic distribution, which suggests that they evolved by duplication from an ancestral sequence that was characteristic for a given group of taxons. On the other hand, some closely related COGs exhibit complementary phyletic distribution (typically: Bacteria and Eukaryota+Archaea) and may be orthologous. This in turn suggests that the SPOUT superfamily had multiple members in the LUCA, which gave rise to the extant COGs only after the radiation of the three Domains of Life. One example of a family that underwent multiple duplications is offered by the 'supercluster', comprising five families with members present in most Bacteria (COG0219, COG0565, and three families of COG0566). The most parsimonious explanation for the results of our analyses is that this group of sequences had a single ancestor in LUCA (most likely the predecessor of extant COG0565), which functioned as a ribose MTase. It was duplicated in the bacterial lineage to yield ancestors of COG0566 and COG0219, followed by further duplications of COG0566 to yield three extant families (the duplication yielding COG566B and COG566C was probably the most recent, as these two COGs share a common fusion with the L30e-like domain). It is likely that all these descendants of the ancestral ribose MTase were transferred to the primordial Eukaryote via the mitochondrial endosymbiont, where virtually all representatives of COG0219 and COG0565 were lost (a single COG0565 member in *E. histolytica *has been most likely reintroduced by HGT from Bacteria), while members of COG0566 were transferred to the nuclear genome and adapted to function in different compartments as well as acquired new functions (e.g. Mrm1p and Trm3p, see above). In parallel, multiple duplications within the archaeal lineage or horizontal transfers from thermophilic Bacteria gave rise to archaeal COGs: 4080, 1818, 1303, and 1772. Alternative scenarios, in which duplications of the ancestral ribose MTase precede LUCA, cannot be completely ruled out, but are in our opinion unlikely. Thus, we predict that all currently known 2'-O-ribose MTases and their homologs identified in this work can be traced to just one ancestor in LUCA.

Another apparently monophyletic pair of families with complementary phyletic distributions is formed by bacterial COG1385 (16S rRNA:m^3^U1498 MTase RsmE) and archaeo-eukaryotic COG2106 (uncharacterized proteins). It is noteworthy that the PUA domain N-terminally fused to the SPOUT domain in COG1385 and the domain inserted into the SPOUT domain in COG2106 both exhibit the same OB-fold, and that they assume a similar spatial orientation with respect to the catalytic domain despite completely different connectivity at the sequence level. It is possible that the unusual architecture of COG2106 has been derived from the tandem domain fusion exhibited by COG1385. The insertion could have been generated in the course of illegitimate recombination, and selected owing to the increased rigidity of the putative RNA-binding OB-fold domain attached to the SPOUT domain by two linkers on both sides. Thus, we predict that COG1385 and COG2106 are orthologous and that their common ancestor was present in LUCA. Since Eukaryota generally lack m^3^U in their SSU rRNA, members of COG2106 could be involved in methylation at a different position than the target of their predicted bacterial orthologs.

The branch comprising m^1^G MTases includes COGs with nearly perfectly complementary phyletic distribution: either exclusively bacterial (with members in plants most likely derived from the bacterial endosymbionts) or exclusively archaeo-eukaryotic. The close relationship between COG2419 and COG2428 suggests that they are paralogs. The exact time of their origin is unclear, as COG2419 is present in nearly all Eukaryota and Archaea, while the presence of COG2428 is restricted to Archaea, fungi, and Plasmodium, suggesting either ancient duplication followed by loss of most members of COG2428 or (in our opinion less likely) a more recent duplication either in Archaea or fungi, followed by GHT to the other lineage (fungi or Archaea, respectively), and to Plasmodium.

The close association between the archaeo-eukaryotic lineage COG2419/COG2428 (tRNAm^1^G9 MTase Trm10) and bacterial COG0336 (tRNA:m^1^G38 MTase TrmD) suggests that they are orthologous branches of an ancient m^1^G MTase family that has been present in LUCA. The ancestral function of members of the entire TrmD/Trm10 clade could have been the N1-methylation of G37 in the anticodon loop, which has been maintained in the very strongly conserved TrmD family (the average sequence identity is higher in COG0336 than in any other SPOUT family). The strong divergence of the COG2419/COG2428 branch could have been caused by the lost competition with Trm5p MTases (members of the RFM superfamily) that functionally replaced TrmD in Archaea and Eukaryota and apparently forced the 'defeated' orthologs of TrmD to migrate into new evolutionary niches that are not occupied in Bacteria, such as such as N1-methylation of G9 in tRNA. COG2419 has been expanded by several duplications in metazoans [52], and its new members have been probably adapted to methylate different substrates (possibly different tRNAs).

The 'm^1^G branch' contains also the bacterial COG1576 (uncharacterized protein YbeA) and archaeo-eukaryotic COG1756, whose relationships to each other and to m^1^G MTases are however unclear. They could either represent another orthologous lineage, whose duplication from the ancestor of m^1^G MTases predated LUCA, or products of more recent, independent duplications of predecessors of the two m^1^G COGs. Duplications predating LUCA followed by lineage-specific loss also cannot be ruled out, however we find them unlikely. Thus, we postulate the most parsimonious scenario, that m^1^G MTases and their homologs evolved from one common ancestor present in or before LUCA. Because of the 'minimal' character of COG1576 both with respect to the number of secondary structures in a protomer and the relatively small dimerization interface, we predict that the Ur-SPOUT protein was structurally similar to YbeA and its relatives.

As mentioned earlier, the relationship of archaeal COG1901 to other families is uncertain. In different analyses it groups together either with m^3^U or m^1^G MTases. However, it does not share the active site with either of these families. We predict it is a product of Archaea-specific duplication, either of COG2106 or one of the m^1^G-lineage families or a derivative of COG1385 horizontally transferred to the ancestor of Archaea from a bacterium. Members of COG1901 may catalyze the formation of Archaea-specific methylation, for instance m^1^Ψ formation, or another modification conserved in most Archaea. Experimental analysis of the bacterial member from *Vibrio vulgaris *(product of an ORF VV21434) may help to resolve this issue.

**Figure 12:**
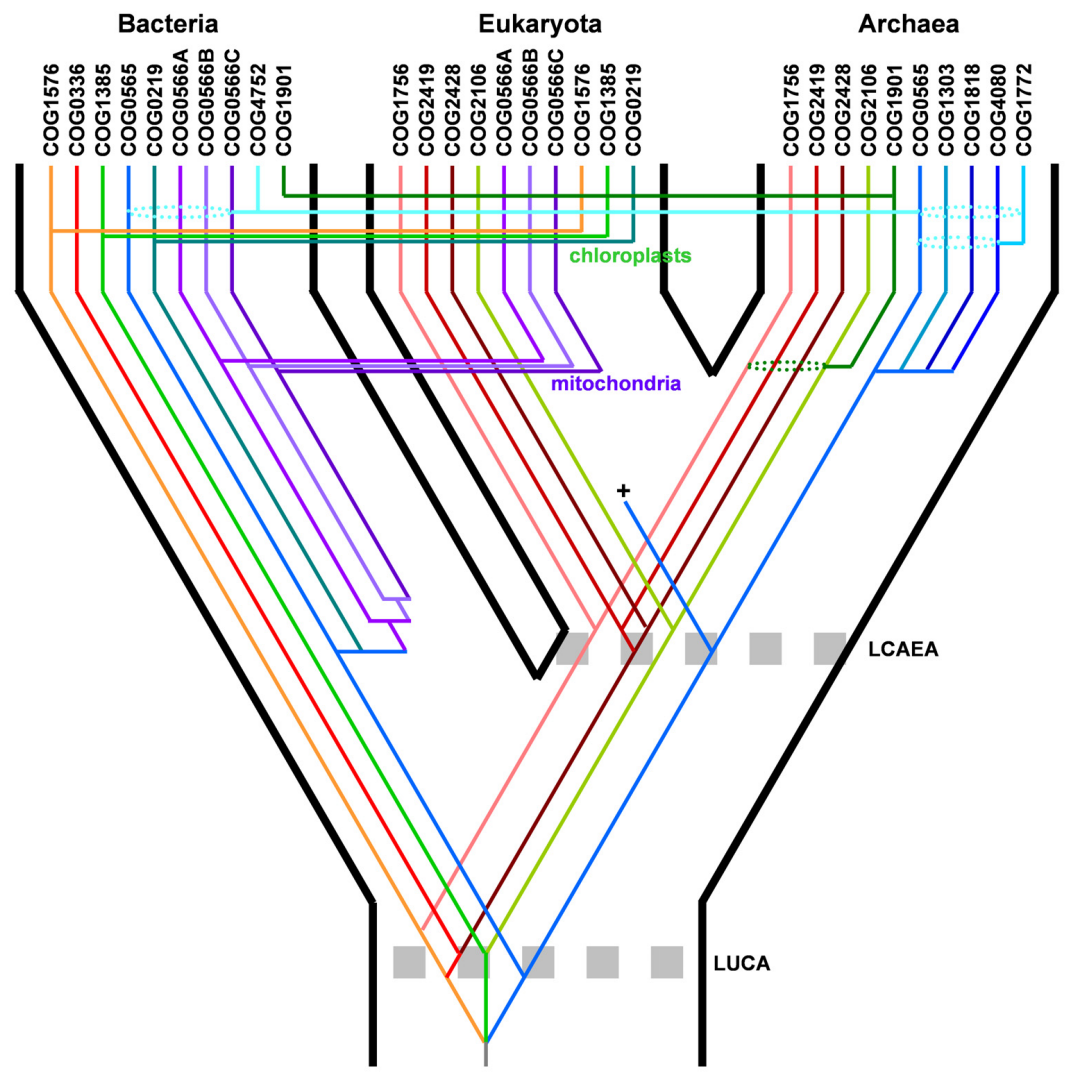
**A speculative scenario of the evolutionary history of the SPOUT superfamily**. This scenario is based on the assumption that Bacteria, Archaea, and Eukaryota are all monophyletic, that Archaea and Eukaryota are sister lineages, and that the root (corresponding to the LUCA) is located in the branch between Bacteria and the Last Common Ancestor of Eukaryota and Archaea (LCAEA). Three major branches corresponding to the three Domains of Life. Lines in different colors indicate COGs (blue: 2'-O-ribose methylation, red: m^1^G methylation, green: m^3^U methylation, grey – unknown). Crosses indicate extinction. Dotted arrows indicate putative horizontal gene transfers. Dashed ellipses indicate uncertainty in the assignment of genes that underwent duplication to yield a particular COG, e.g. they encompass sets of potential mother lineages.

Summarizing, we predict that LUCA contained at least three SPOUT members, involved in ribose 2'-O-methylaton, guanosine N1-methylation, and uridine N3-methylation (or a similar reaction, such as methylation of endocyclic nitrogen atoms in another base). Our speculative scenario of the evolutionary history of the SPOUT superfamily is illustrated in Figure 12.

### Before the LUCA: Speculations on the possible origin of the α/β knot in the SPOUT superfamily

The SPOUT superfamily is unique in that all its characterized members possess a very deep knot. The only exception is TrmD from *A. aeolicus*, in whose structure (1oy5) no knot has been observed [63]. However, comparison with very closely related, independently determined and published about the same time structures of TrmD from *H. influenzae *(1uak) [10] and *E. coli *(1p9p) [11] reveal that its lack of the knot may be an artifact of crystallographic data interpretation. The 'unknotted' loop in 1oy5 blocks the AdoMet-binding site, which suggests that it was probably misthreaded into the electron density of the cofactor (data not shown). Amino acid residues involved in stabilization of the knot are conserved in the TrmD family, therefore we believe that the true structure of *A. aeolicus *TrmD is knotted as well.

Despite the conservation of the knot among SPOUT superfamily members, virtually nothing is known about its origin, both in the time scale of evolution, when the SPOUT ancestor appeared, and in the time scale of protein folding. Experimental analysis of the YibK protein revealed that it can be denatured reversibly, and that it folds via two parallel pathways partitioned by proline isomerization events, to two distinct monomeric intermediates with native-like secondary and tertiary structure [64,65]. However, the mechanism of formation of the knot itself has not been analyzed. The analysis of protein dynamics to identify potential folding pathways of SPOUT proteins is beyond the scope of this study. Nevertheless, we would like to point out that the presence of fusions of SPOUT domains with other large domains at both termini, and in particular tandem fusions of SPOUT domains within the same polypeptide identified in this work (Figure 3), suggests that either the knotting must involve very large loops, through which the whole domains are threaded, or that knot formation may occur in either direction, i.e. by threading of either N- or C-terminal regions. The detailed analysis of the SPOUT folding pathways must however await additional experimental data.

**Figure 13:**
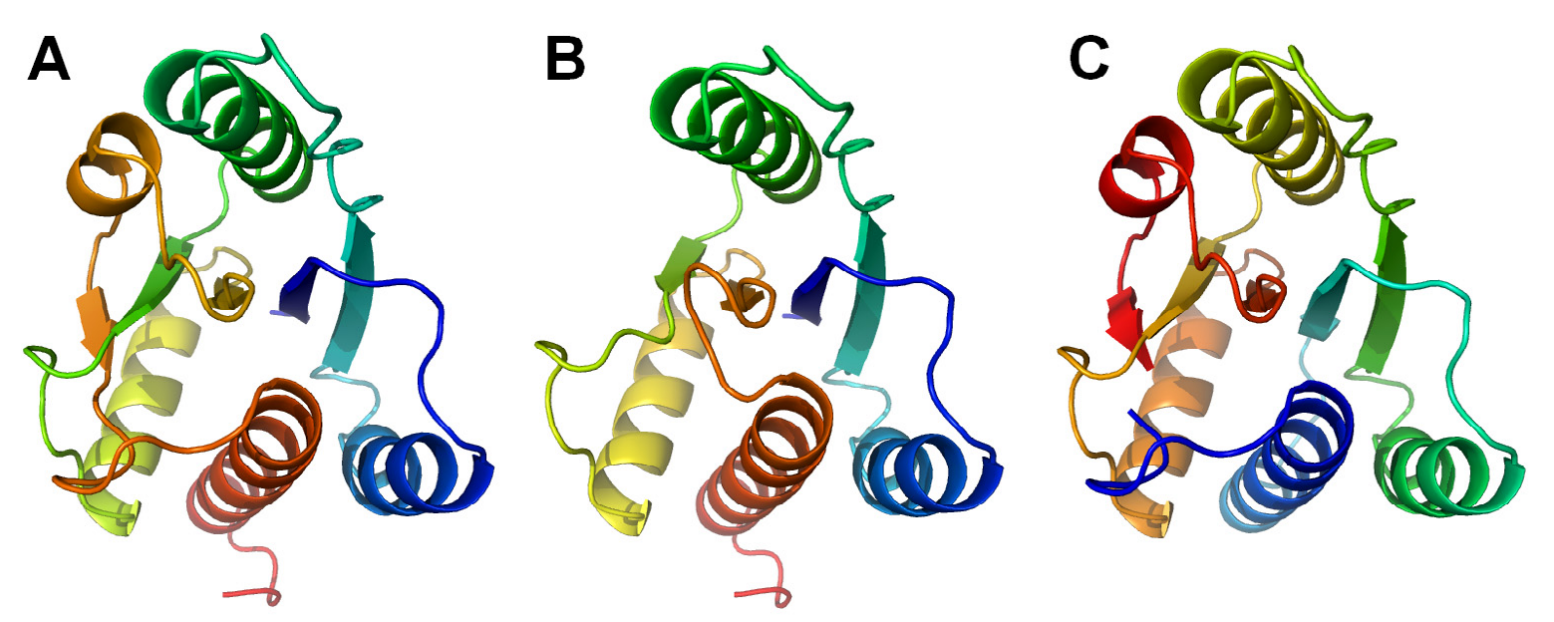
**Possible origins of the knot**. A) Contemporary knotted SPOUT fold, exemplified by *E. coli *YbeA (1ns5). B) Putative ancestral structure, 'unknotted' by deletion of an α/β unit. C) Alternative putative ancestral structure, 'unknotted' by circular permutation, i.e. linking 'old' termini and cutting the knot to create new termini. Protein sequence is colored from blue (N-terminus) to red (C-terminus).

Virnau and co-workers have recently examined the occurrence and conservation of knots in structures in the Protein Data Bank [66]. Apart from the already known SPOUT MTases with a conserved knot, they found a pair of proteins from a different superfamily, for which topology is not preserved. N-acetylornithine transcarbamylase (AOTCase) contains a deep trefoil knot similar to that in SPOUT MTases (a C-terminal α-helix threaded through a loop connecting a β-strand with another α-helix) [67], while its close homolog L-ornithine transcarbamylase (OTCase) is unknotted [68]. Despite the different topology, both structures have the same fold, and their backbone coordinates are nearly identical and differ mainly in the length of the loop that forms the knot. The most parsimonious explanation is that a short insertion enlarged the loop, thus allowing for threading the C-terminal helix through that loop before the rest of the protein is fully folded [66]. That simple mechanism suggests an analogous scenario for the origin of the deep knot in the SPOUT superfamily. We hypothesize that a region preceding the knotted C-terminal helix, including the AdoMet-binding loop, a β-strand, and the 2^nd^α-helix from the C-terminus, originated from an insertion in the ancestral Rossmanoidal fold, comprising originally probably only 4 β-strands and 3 α-helices (Figure 13). The insertion was longer than in AOTCase, and formed not just a loop, but a new αβ segment that extended the core sheet, as frequently happens in the evolution of α/β proteins [69-71]. Hydrogen-bonding of the new strand to the β-sheet forced the C-terminal helix to pass below the arch formed by the loop at the new edge of the β-sheet, thereby forming the knot. A part of the insertion formed a new loop that in extant SPOUT MTases serves as a cofactor-binding site. Binding of the cofactor might have provided stability to the rearranged structure, as well as a new function (methylation). The creation of the knot and/or the cofactor binding site probably required secondary adaptive mutation which stabilized the unique structure of the ancestral SPOUT fold. Some of these adaptations could have involved dimerization and/or RNA binding, although it cannot be excluded that these features were already present in the ancestral unknotted protein. Regrettably, we could not detect any known protein structures with the same fold as our hypothetical pre-SPOUT Rossmanoid. Therefore, we cannot propose any detailed hypothesis about its function or relationship to the known folds, besides the very general similarity to Rossmanoidal proteins that often bind nucleosides.

An alternative mechanism for the knot formation in the SPOUT fold involves an un-knotted ancestor with a similar number and spatial orientation of secondary structure elements to those present in the extant SPOUT MTases, albeit with a circularly permuted order of these elements. Circular permutations have been reported in various DNA and RNA MTases from the RFM superfamily [72-74]. It has been speculated that permutation could have played a role in the evolution of new specificities and appearance of dimerization in DNA MTases [75,76]. In the SPOUT domain the N- and C-termini are adjacent in space, suggesting that the C-terminal helix could be covalently linked to the N-terminal strand without the need of a long linker. However, the introduction of new termini in the place of the knot would lead to the interruption and possibly destabilization of the AdoMet-binding loop. Thus, circular permutation from an unknotted ancestor with the AdoMet-binding site formed by flexible termini could have been selected for the improved cofactor binding and catalytic efficiency.

The aforementioned hypothetical scenarios for the origin of the SPOUT fold can be tested experimentally, e.g. by reconstruction of the putative insertion-less and circularly un-permuted ancestral SPOUT structures and studies of their stability, ability to form dimers, ability to bind AdoMet and/or RNA, and the enzymatic activity. The comparative analysis of the SPOUT superfamily presented in this work will help selecting optimal targets for such experiments and will aid in understanding of the determinants for formation, stability and function of the knots.

## Discussion

The availability of the complete catalog of RNA MTases and understanding the mechanism of their action on the biochemical and cellular level is an essential stepping stone for the complete understanding of mechanisms that govern posttranscriptional modification and maturation of RNA. Thus far, only a fraction of known methylations sites in RNA has been linked to known protein families. Among several superfamilies of MTases, whose substrates include RNA, SPOUT enzymes are unique in that none of them has been ever found to act on any other substrate than RNA. This preference for one type of macromolecular substrate makes the newly discovered members of the SPOUT superfamily very attractive candidates for functional analyses. However, since the pioneering analysis of Anantharaman, Koonin, and Aravind, who identified homology between SpoU and TrmD MTases and defined the SPOUT superfamily [1], no comprehensive comparative analysis of these proteins has been published. Since then, many crystal structures of SPOUT-fold proteins have been solved and numerous new sequences appeared in the databases. Many of the structures were solved by structural genomics centers, which provide little, if any functional information (Table 2).

Motivated by the availability of numerous poorly annotated structures and sequences of apparent SPOUT homologs and the lack of a good 'road map' to navigate between them, we carried out extensive bioinformatics analyses aimed at comprehensive classification of the SPOUT superfamily and making functional predictions for the emerging (sub)families of uncharacterized proteins. Our results revealed new members of previously identified SPOUT families, and suggested that several previously identified, but structurally uncharacterized protein families also belong to the SPOUT superfamily. In particular, we predict that the COG4080 family annotated as RecB nuclease homologs should be reclassified as putative MTases, and that the COG1756 family of proteins implicated in rRNA maturation and indirectly related to methylation may be catalytically active as MTases. It must be mentioned, however, that MTase homologs may exhibit non-MTase functions. There is a number of MTase-like members of the RFM superfamily, which bind AdoMet (or similar cofactors), but catalyze different reactions, such as methylene transfer, condensation of AdoMet, transfer of the aminopropyl groups, or hydroxylation. Moreover, a number of RFM proteins have been reported to be enzymatically inactive, but involved in other activities e.g. nucleic acid binding (review: [5]). So far, this phenomenon has not been reported for SPOUT MTases, but in principle it cannot be excluded that some of the uncharacterized SPOUTs analyzed in this article carry out different reactions than methylation (with or without AdoMet) or are enzymatically inactive. As mentioned in the Results, one candidate for an inactive, regulatory or substrate-binding subunit of a heterodimeric complex is the Ymr310cp protein, a close paralogs of Ygr283cp (COG 2106).

## Conclusion

We delineated the common core of SPOUT MTases and inferred a multiple sequence alignment for the conserved knot region, from which we calculated the phylogenetic tree of the superfamily. We also analyzed the phylogenetic distribution of different families, and used this information to infer the evolutionary history of the SPOUT superfamily and the number of ancient SPOUT enzymes in LUCA. We also proposed tentative scenarios for the origin of the deep knot in the SPOUT fold. In parallel, we correlated the presence of SPOUT members with the presence of known RNA modifications in *E. coli *for which no enzymes have been assigned yet. Based on the conservation of the putative active sites, we predicted biochemical functions for some of the so far uncharacterized families. These predictions will guide experimental analyses aiming at better understanding sequence-structure-function relationships in the SPOUT superfamily, as well as completing the picture of RNA modification pathways. Detailed characterization of all SPOUT MTases from model organisms may be the first important step towards this goal.

## Methods

### Sequence database searches, multiple alignments and comparison of sequence-structure profile HMMs

Representative members of previously identified families within the SPOUT superfamily (TrmH (SpoU), GI:48425869; RlmB (YjfH), GI:24987276; YsgA, GI:22218790; LasT, GI:12516936; YibK, GI:466744; PF0461, GI:18976833; TM1570, GI:15644318, PF1826, GI:18978198; TrmD, GI:57240404; AF2226, GI:11499808; Trm10, GI:39584992; RsmE, GI:18313154; YbeA, GI:28374103; PF1588, GI:18977960) were used as seeds in PSI-BLAST [77] searches of the non-redundant (nr) version of current sequence databases (nr) and the publicly available complete and incomplete genome sequences with the expectation (e) value threshold for the retrieval of related sequences set to 10^-3^. All retrieved sequences were subsequently realigned using MUSCLE [28,78] to the degapped profiles obtained from the multiple sequence alignments reported by BLAST. Additional sequence alignments were done with MUMMALS [79]. Incomplete sequences were discarded (if the deletion spanned > 30% of the alignment) or repaired using amino acid sequences predicted from the available DNA sequences of the corresponding genes. Manual adjustments were introduced into the alignments to preserve the continuity of secondary structure elements, either observed in crystal structures or predicted computationally (see below). The alignments were used to generate a set of query profile-HMMs using HHmake from the HHsearch package [25] and searched against the profile-HHMs corresponding to alignments of protein families obtained from the COG, KOG [80], and PFAM [81] databases. The comparison of the profile HMMs (including protein sequences and consensus secondary structure) was carried out using HHsearch [25], with default parameters. Results of the most recent CASP7 competition (announced while this article was under review) demonstrated an outstanding performance of HHsearch (as a HHpred server) in remote protein homology detection, which validates our choice of this method as the primary tool for the analysis of the SPOUT superfamily.

### Sequence clustering

To identify (sub)families of closely related sequences and visualize similarities within and between all SPOUT members we used CLANS (CLuster ANalysis of Sequences), a Java utility that applies version of the Fruchterman-Reingold graph layout algorithm [27]. CLANS uses the P-values of high-scoring segment pairs (HSPs) obtained from an N × N BLAST search, to compute attractive and repulsive forces between each sequence pair in a user-defined dataset. A 3D or 2D representation is achieved by randomly seeding sequences in the arbitrary distance space. The sequences are then moved within this environment according to the force vectors resulting from all pairwise interactions and the process is repeated to convergence.

### Structural alignment of SPOUT families

The representative SPOUT structures (Table 1) were first superimposed using SwissPDBViewer [82]. The automatically generated sequence alignment based on this superposition was carefully analyzed and verified manually, to identify all homologous positions and maximize the number of aligned residues between as many structures as possible, provided that there was a rational basis for the alignment. At all times, the alignment was guided by direct visual inspection of the structures. The resulting high-quality alignment was used as a starting point to add computationally modeled structures for those families, which lacked experimental structural information. In a few cases, the confrontation of the multiple structure alignment has lead to slight revisions of the model (e.g. rebuilding it with small residue shifts). Because of significant structural divergence of the N-terminal Rossmanoidal part of the SPOUT fold and the uncertainty of many models in this region, we decided to retain only the knotted part of the structure, comprising four strands and two helices. As a result, we obtained a high-quality multiple sequence alignment of a structurally conserved region common to all SPOUT proteins, comprising at least one representative from each family analyzed in this work. Subsequently, we 'extended' this alignment by adding more members for each family, based on sequence alignments constructed independently for each family. We believe this alignment is of sufficient quality to serve as a 'gold standard' for studying the SPOUT superfamily.

### Phylogenetic analyses

MrBayes MPI version 3.1.2 [58] was used to carry out a Bayesian analysis of data. A combined analysis was preformed using both the sequence alignment for family representatives (only the confidently aligned knotted region) and features as defined in Table 2. Features were encoded as standard unordered characters. The sequence dataset was modeled with a gamma distribution of substitution rates, using the default approximation of four rate classes for each. Preliminary runs with MrBayes using a mixture of model priors demonstrated conclusively that priors based on the substitution rates from the BLOSUM matrices [83] provided best fit to the sequence data. Therefore, the BLOSUM model was used to provide substitution priors for the sequence partition of the data. A Metropolis-coupled Markov-chain Monte-Carlo analysis was preformed with 2.000.000 generations, two runs and eight chains (four per run). The Markov chain was sampled every 100 generations. Convergence of runs was confirmed by average standard split deviation factor that falls under the recommended value of 0.01. The final tree was obtained after removing the first 25% of samples. Analogous calculations were carried out for the sequence alignment and the feature matrix alone. These runs used identical parameter settings to those for the mixed model for the corresponding datasets.

The phylogenetic tree for the supercluster was inferred for 200 representative members of COGs 0219, 0565, and 566, using the alignment of a complete catalytic domain (not only the knotted part, but also the Rossmanoidal N-terminus). Representative members were selected from all members of the supercluster using the 'purge' option of the Gibbs sampler [84], with the BLOSUM62 score of 300 as a threshold for maximal sequence similarity to include any two sequences in the output file. A Minimum Evolution analysis carried out with MEGA 3.1 [85] (with pairwise gaps deletion and either Dayhoff or JTT matrices) was sufficient to infer a tree with pre-defined subfamilies grouped into monophyletic branches, thus the Bayesian analysis was not attempted. Since the bootstrap test tends to become progressively more conservative as the number of sequences in the tree increases, we assessed the stability of the tree using the interior branch test [86], which is believed to have virtually the same statistical properties as the bootstrap test irrespective of the number of sequences used.

### Protein structure prediction

Secondary structure prediction and tertiary fold-recognition was carried out via the GeneSilico meta-server gateway [26]. Secondary structure was predicted using a consensus of PSIPRED [87], PROFsec [88], PROF [89], SABLE [90], JNET [91], JUFO [92], PORTER [93], SSPRO2 [94] and SAM-T02 [95]. Solvent accessibility for the individual residues was predicted with SABLE [90], ACCPRO2 [94], and JNET [91]. The fold-recognition (FR) analysis (attempt to match the query sequence to known protein structures) was carried out using PDBBLAST (local implementation of a PSI-BLAST [77] search against sequences of proteins from the PDB), HHSEARCH [25], FFAS03 [96], FORTE [97], SAM-T02 [95], 3DPSSM [98], INBGU [99], FUGUE [100], mGENTHREADER [101], and SPARKS [102]. Target-template alignments reported by these methods were compared, evaluated, and ranked by the PCONS server [103] to identify the preferred modeling templates and the consensus alignment.

### Homology modeling and model assessment

The alignments between the sequences of the selected representatives of all COGs of unknown structures and the structures of selected templates identified by Pcons were used as a starting point for modeling of the members of: COG0565, COG4752, COG1303, COG1818, COG1772, COG4080, COG1901, COG2419, COG2428 and COG1756 tertiary structure using the "FRankenstein's Monster" approach [104,105], comprising cycles of model building, evaluation, realignment in poorly scored regions and merging of best scoring fragments. The positions of predicted catalytic residues and secondary structure elements were used as spatial restraints. This methodology was described in great detail in a number of our previous papers on MTases and other enzymes, the readers are encouraged to check out the recent articles describing the modeling of RNA MTases TrmJ [30] and HEN1 [106]. The 'FRankenstein's monster' approach was objectively found as one of the most accurate methods for template-based modeling of protein structures in the rankings of CASP5 and CASP6 [107,108]. For the evaluation of models we used PROQ [109,110], and a MetaMQAP method recently developed in our group [111], which allow predicting the deviation of individual residues in the model from their counterparts in the native structure. Models were evaluated and corrected if certain regions were not modeled confidently (e.g. we were not certain of the alignment), and exhibited poor PROQ or MetaMQAP scores.

In this analysis, modeling had only a supporting role for the generation of high-quality alignments, and regions that could not be modeled reliably were left 'unfinished'. Therefore, the models of individual proteins are not shown. They can be, however, obtained from the corresponding author (J.M.B.) upon request.

## List of abbreviations

aa, amino acid(s); bp, base pair(s); nt, nucleotide; e, expectation; MTase, methyltransferase; ORF, product of an open reading frame, RFM, Rossmann-fold MTase; AdoMet, S-adenosyl-L-methionine.

## Authors' contributions

KLT carried out sequence and structure database searches, prepared alignments, structural superpositions, performed homology modeling, participated in preparation of the figures, interpretation of results and drafted the manuscript. SD-H carried out Bayesian phylogenetic analyses and participated in interpretation of the results. SD-H and EP carried out sequence searches and domain analysis of the 'supercluster' (COGs 0219, 0566, and 0565). JMB coordinated the whole study, participated in all analyses, interpreted the data, and prepared the final version of the manuscript. All authors have read and accepted the final version of the manuscript.
